# Application of Nano-Delivery Systems in Lymph Nodes for Tumor Immunotherapy

**DOI:** 10.1007/s40820-023-01125-2

**Published:** 2023-06-03

**Authors:** Yiming Xia, Shunli Fu, Qingping Ma, Yongjun Liu, Na Zhang

**Affiliations:** Department of Pharmaceutics, Key Laboratory of Chemical Biology (Ministry of Education), NMPA Key Laboratory for Technology Research and Evaluation of Drug Products, School of Pharmaceutical Sciences, Cheeloo College of Medicine, Shandong University, 44 Wenhua Xi Road, Jinan, 250012 Shandong People’s Republic of China

**Keywords:** Cancer therapy, Immunotherapy, Lymph nodes, Nano-delivery systems

## Abstract

Immunotherapy has become a promising research “hotspot” in cancer treatment. “Soldier” immune cells are not uniform throughout the body; they accumulate mostly in the immune organs such as the spleen and lymph nodes (LNs), etc. The unique structure of LNs provides the microenvironment suitable for the survival, activation, and proliferation of multiple types of immune cells. LNs play an important role in both the initiation of adaptive immunity and the generation of durable anti-tumor responses. Antigens taken up by antigen-presenting cells in peripheral tissues need to migrate with lymphatic fluid to LNs to activate the lymphocytes therein. Meanwhile, the accumulation and retaining of many immune functional compounds in LNs enhance their efficacy significantly. Therefore, LNs have become a key target for tumor immunotherapy. Unfortunately, the nonspecific distribution of the immune drugs in vivo greatly limits the activation and proliferation of immune cells, which leads to unsatisfactory anti-tumor effects. The efficient nano-delivery system to LNs is an effective strategy to maximize the efficacy of immune drugs. Nano-delivery systems have shown beneficial in improving biodistribution and enhancing accumulation in lymphoid tissues, exhibiting powerful and promising prospects for achieving effective delivery to LNs. Herein, the physiological structure and the delivery barriers of LNs were summarized and the factors affecting LNs accumulation were discussed thoroughly. Moreover, developments in nano-delivery systems were reviewed and the transformation prospects of LNs targeting nanocarriers were summarized and discussed.

## Highlights

- The physiological structure and the drug delivery barriers of lymph nodes were described.
- The factors affecting lymph nodes accumulation in nano-delivery systems were discussed.
- The recent progress of nano-delivery carriers applied for lymph nodes immunotherapy was further categorized and reviewed.

## Graphical abstract

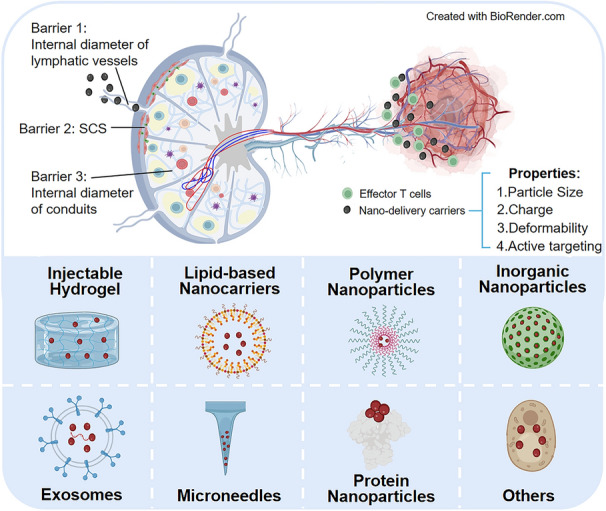

## Introduction

Immunotherapy has shown great promise in clinical tumor treatment [1], which could enhance the anti-tumor immune response by increasing the immunogenicity of tumor cells and the sensitivity to immune cells or combining the components of the immune system to inhibit tumor growth [2, 3]. Currently, immunotherapy mainly includes tumor vaccines, immune checkpoint inhibitors, and chimeric antigen receptor (CAR) T-cell therapy [4, 5], which shows great clinical effects. The first therapeutic vaccine for prostate cancer was approved by the Food and Drug Administration (FDA) in 2010, followed by the CTLA-4 antibody (Ipilimumab) and PD-1 antibody (Nivolumab) in 2011 and 2014, respectively [6], then the CAR-T cell therapy Kymriah and Yescarta were approved by the FDA in 2017 [7]. Now immunotherapy has become the mainstay of tumor treatment. Although immunotherapy has made a great breakthrough in tumor therapy, the low response rate and the immune-related side effects have limited its application in the clinic [8, 9].

Effective immune cell activation requires the precise delivery of immune functional compounds to the target immune cells. However, immune cells are not uniformly distributed throughout the body and are mainly located in the secondary lymphoid organs such as the spleen and LNs [10]. Secondary lymphoid organs play an important role in establishing and regulating the immune response, which is crucial for tumor development and immunotherapy. LNs are the critical station for the collection of tumor antigens and the priming of the immune response. Specifically, the antigen-presenting cells (APCs) could recognize and present tumor antigens from the surrounding tissues and then deliver them to the LNs. Besides, for the free antigens, the complement or antibodies could combine with them and then enter the LNs through the lymphatic fluid [11]. Subsequently, immune cells within LNs will recognize the antigen and activate the antigen-specific T cells to initiate adaptive immunity. Then tumor antigen-specific cytotoxic T cells (CTLs) are produced and transported to the tumor site for killing action [12]. A growing number of researches suggest that LNs may be a key therapeutic target for emerging tumor immunotherapy and the LNs-targeting delivery system is a key means to modulate the immune response to promote immune cell activity.

Nano-delivery systems offer the possibility of effective LNs delivery [13]. Nanomedicines have gradually become a new favorite in the pharmaceutical field, influencing the original drug development paradigm. It is expected that the nanomedicine market may reach $350.8 billion by 2025 [14]. Nano-delivery systems have highly controllable physicochemical properties that could increase the molecular weight of the delivered drug, improve the undesirable properties of the delivered drug, enhance its permeability, and improve its tissue distribution and in vivo metabolism [15–17]. In addition, targeted drug distribution could be achieved through flexible surface modifications, such as the use of APCs targeting ligands in peripheral tissues to facilitate their transport to lymphoid tissues, which could increase the locally high accumulation of the drug in the LNs [18] for improving the anti-tumor effects and reducing the toxic side effects. Hence, the nano-delivery system shows beneficial dynamic interactions with lymphoid organs and promising applications for efficient LNs delivery.

Based on the critical role of LNs in the anti-tumor immunotherapy, the physiological structure and the delivery barriers of the nanodrug delivery system are summarized and the innovative applications of the nano-delivery system in LNs are reviewed and discussed, indicating the direction of optimization and breakthrough for LNs immunotherapy delivery system.

## Structure of LNs

**Fig. 1:**
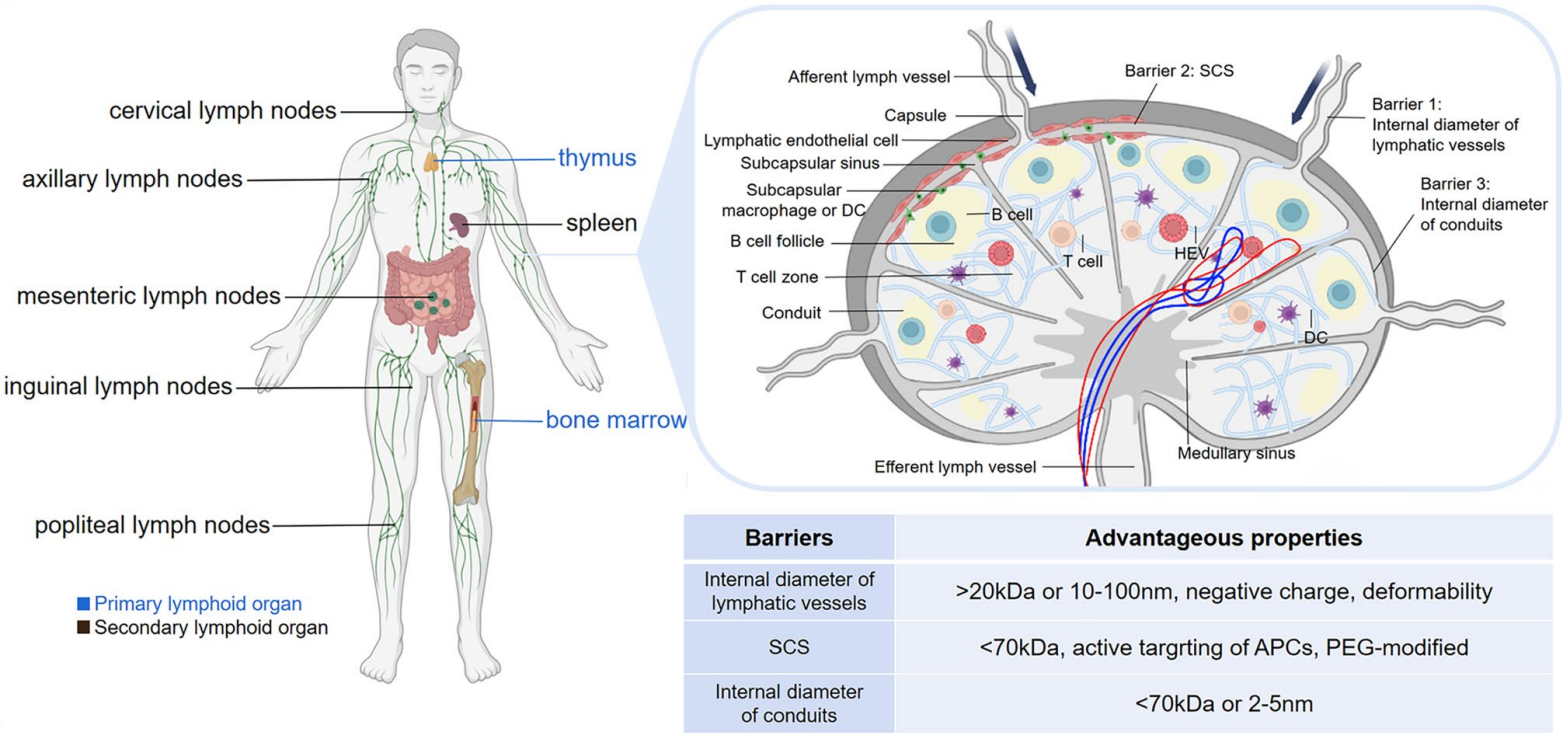
Schematic diagram of LNs distribution, structure and barriers. Created with BioRender.com

The lymphatic system plays an important role in maintaining humoral homeostasis and regulating adaptive immune responses [19]. Primary lymphoid organs, such as the thymus and bone marrow, are sites where hematopoietic stem cells proliferate and differentiate into prolymphocytes. Then, lymphocytes are transported to secondary lymphoid organs by L- selectin binding to peripheral lymph node vascular addressin (PNAd) [20, 21]. Secondary lymphatic organs include the spleen, LNs, peyer's patches (PPs), tonsils, etc., which are the sites for the settlement and proliferation of various immune cells as well as important parts of the immune response [22]. LNs are widely distributed in the body and mainly include cervical, axillary, mesenteric, inguinal, and popliteal lymph nodes (PLNs) [23]. Understanding the physiological structure of LNs and clarifying the barriers of the nano-delivery system is the precondition for designing efficient and rational nano-delivery systems (Fig. 1).

LNs are the “home base” of immune cells (APCs, T cells and B cells, and so on) [24, 25], which are wrapped by a thin fibrous capsule and could recognize the antigen signal through the afferent lymphatics [26]. Under the capsule, there are barrier cells such as macrophages and others constructed in the subcapsular sinus (SCS) [27]. Studies have shown that depleting SCS macrophages allows more nanoparticles to access LNs [28]. Beneath the SCS is the cortex, which contains narrow conduits of 3–5 nm in diameter [29]. For free antigens, molecular weight is a critical factor to determine their passage through the SCS. Large molecules (≥ 70 kDa) are confined outside the LNs conduits and cortex by the SCS [18, 30], so they are required APCs, such as dendritic cells (DCs), macrophages and migrating B cells, to transport into the interior of the LNs. Small molecules (< 70 kDa or 2–5 nm) could directly enter the conduits. In contrast, APCs that recognize antigens in peripheral tissues and migrate into LNs exhibit other mechanisms, which are mainly dependent on the concentration gradient of chemokines [31, 32]. Upon antigen stimulation, APCs become highly expressed on the surface of chemokine ligands CC-chemokine ligand 7 (CCR7), which penetrates deeply into the lymphatic vessels under the highly expressed chemokine ligands CC-chemokine ligand 21 (CCL21) [33, 34]. The lymphatic endothelial cells specifically express atypical chemokine receptor 4 (ACKR4), which is capable of scavenging CCL21, thus creating a local gradient of CCL21, so that APCs can penetrate deeper into LNs under the chemotactic effect of CCL21 [12, 35].

Inside the LNs are mainly divided into the cortical area and the medulla area, with the capsule connective tissue extending towards the parenchyma to form trabeculae [36]. Cortical areas can be structurally divided into the cortex and paracortex [37]. The cortex is the main area where B cells settle and contain spherical follicles [20, 37]. The primary follicles are not stimulated by antigens and have no germinal centers in the spherical follicles [18]. After antigen stimulation, follicular dendritic cells (FDCs) can capture antigens for cross-linking B cells for antigen delivery. Then, these B cells proliferate in lymphoid follicles and form germinal centers, called secondary follicles. After proliferation, some of the B cells become plasma cells and could leave the germinal centers [25, 27]. Paracortex is located below the follicle and above the medulla, which is mainly the site of T cells settlement and scattered with DCs and macrophages. Migrating conventional type 1 dendritic cells (cDC1s) and conventional type 2 dendritic cells (cDC2s) could carry most of the antigens into the draining LNs, transfer the antigen via vesicles to resident DCs, thus initiating T-cell based immunity [32]. In addition, many postcapillary microvenules composed of endothelial cells, also known as high endothelial venules (HEVs), are present in the paracortex. HEVs are discontinuously distributed and allow the passage of lymphocytes, serving as a gate for B and T cells entering the LNs from the blood. It is an important pathway to communicate between the blood and lymphatic circulation [27, 36]. Medulla consists mainly of the medullary cords, medullary sinus, and numerous lymphatic vessels. The medullary cords are dense aggregates of lymphocytes, mainly B cells and plasma cells, but also contain some T cells and macrophages. The medullary sinus is rich in macrophages, which have a strong ability to capture and remove pathogens [37, 38].

In conclusion, the physiological structure of LNs provides three barriers to the targeted delivery of nano-delivery carriers. Firstly, the internal diameter of the lymphatic vessels allows only appropriately sized particles to pass through, and the negative charge and deformability of nanoparticles facilitate migration in the lymphatic vessels. Second, macrophages in SCS clear larger sized particles from the lymph fluid, which can be avoided by PEG modification. Or larger particles can be presented by DCs, so the active targeting to APCs can promote the nanoparticles into LNs. Third, the very small internal diameter of the conduits presents a challenge in reaching the T and B cells regions of the LNs. Therefore, nanoparticles need to have appropriate properties for successful accumulation in LNs.

## Administration Routes towards LNs

The exchange of information and substances between LNs and other tissues relies on the blood and lymphatic systems by afferent lymphatics, efferent lymphatics, and abundant HEVs [27]. So effective LNs delivery could be achieved by intranodal injection, oral administration, intravenous administration, and interstitial administration.

Intranodal injection is undoubtedly one of the most effective ways to achieve effective LNs accumulation, especially for delivering particles with limited mobility in the extracellular matrix [23]. Intranodal injection has been applied in the LNs-targeted delivery, because it can achieve better accumulation in the LNs and elicit strong immune responses at low doses [24]. However, LNs are cryptic and structurally fragile [39], which greatly limits their application in the clinic because of the difficult intranodal injection technology. Currently, intranodal injection needs to be performed in conjunction with surgery, ultrasound, or non-toxic tracer dyes, which provides a potential risk for the precise delivery of LNs [40, 41]. In addition, LNs are very tiny tissues with limited dose accommodation for administration, which imposes stringent requirements on the dose and volume to be delivered [42]. Therefore, intranodal injection is not an ideal route of drug administration in practice. However, many LNs-targeted vaccines use intranodal injection because it can avoid the loss of vaccine antigen during delivery [43–45]. For example, JEWELL C M et al. [46] combined intranodal injection vaccination with controlled release biomaterials for adjuvant delivery, and finally succeeded in promoting sustained activation of DCs and amplifying humoral responses.

The capillary wall and the capillary lymphatic wall are the barriers that must be overcame for intravenous administration. Theoretically, the nano-delivery system is not conducive to LNs targeting, because intravenous drugs need to penetrate tissue space through capillaries to be absorbed through lymphatic vessels. On the one hand, the presence of the mononuclear phagocyte system (MPS) recognizes nanoparticles as “foreign bodies”, which leads to non-specific clearance from the blood stream [19]. Adjusting the size and charge of nanoparticles and other properties can enhance nanoparticle circulation [47, 48]. On the other hand, nanoparticles need to extravasate out of the blood vessel endothelium, and diffuse past the extracellular matrix (ECM) in the interstitium. This process can be hindered by vascular endothelial tight junctions and the collagen matrix in the extracellular matrix [49, 50]. Because of the relatively low efficiency of LNs delivery by intravenous administration [51], the use of intravenous administration is currently limited. Intravenous administration mainly focuses on the diagnosis or treatment of tumor LNs metastasis [51, 52]. For example, Xia [53] et al. demonstrated the intravenous mode of indocyanine green (ICG) administration has a higher diagnostic value for localization of metastatic LNs than peritumoral injection in clinical samples of head and neck squamous cell carcinoma (HNSCC).

Oral administration is the most widely used in the clinic. Because the blood flow velocity of mesenteric vessels is 300–1000 times higher than that of lymphatic fluid, combined with the complex physiological environment and physiological barrier of the intestine, it is difficult to achieve lymphatic accumulation of drugs with oral low molecular weight drug solutions [54, 55]. Combining drugs with nanodrug delivery carriers is an effective strategy to achieve oral LNs delivery. M cells in Peper's LNs presented in the intestine can capture particles such as pathogens from the intestinal lumen and transport them to the pocket on the other side of the M cells for APCs residing there to take over [54, 56]. Therefore, the construction of nano-delivery systems targeting M cells is a promising delivery strategy for LNs [57]. For nanoparticles, intestinal uptake depends largely on the physicochemical properties of the particles [58]. In general, particles of nanometer to low micron size may be more efficiently absorbed by M cells or epithelial cells. However, it is not clear whether oral nanoparticles can be absorbed through the intestinal lymphatic system and reach the therapeutic load. The researchers thought that the bioavailability of nanoparticles varies greatly under different conditions [54]. In addition, lipophilic drugs can bind to lipoproteins, the size of which makes crossing the vascular endothelium restricted, leading to the preferential entry of drug-lipoprotein complexes into the intestinal lymphatic system [59]. The process can be facilitated by modified pre-drugs with an affinity for lipoproteins, co-administered with lipid-derived foods or preparations [55]. At present, the oral cancer vaccine is a hot research direction. Recombinant or attenuated strains of various bacteria are often used as carriers for oral vaccines because they can deliver antigens into the gut-associated lymphoid tissue [57]. Hu et al. [60] took advantage of the ability of oral Salmonella to colonize intestinal associated lymphoid tissues via Peyer's patches. They coated polymer nanoparticles containing DNA on attenuated Salmonella carriers. Experiments demonstrated that this oral DNA vaccine had efficient phagosome escape and enhanced acid resistance. However, it has also been shown that lymphatic uptake of orally delivered nanoparticles is minimal [18].

Interstitial administration is a local delivery method that includes intramuscular, subcutaneous, intradermal, or intra-tumoral tissue interstitial administration [61]. Interstitial administration is the main mode of administration routes towards LNs currently. After interstitial administration, the choice of nanoparticles to be transported through capillaries or capillary lymphatics depends on the nature of the nanoparticles. For example, nanoparticles of a few microns mostly enter the capillaries. Nanoparticles in 10–100 nm can enter the LNs through the capillary lymphatics. For nanoparticles larger than 100 nm, they are trapped in the interstitium and processed by DCs to reach the LNs [18, 26]. In the case of interstitial administration of lymphatic-targeted vaccines, antigens are captured by APCs in peripheral tissues, and cross the lymphatic endothelium from the interstitium into the interstitial fluid and migrate to LNs in the presence of chemokines [24]. Interstitial fluid pressure and flow are also factors affecting lymphatic transport, and co-administration with substances that can increase interstitial pressure is expected to promote lymphatic uptake [54, 62, 63]. In addition, the return of albumin from the interstitial fluid to the circulation through the lymphatics, in response to "albumin hitchhiking", an endogenous protein for targeted transport, is a promising strategy [64].

In summary, the most promising administration route towards LNs is interstitial administration at present. This is consistent with the administration route of LNs targeted nano-delivery systems in recent years.

## Nanosystem Properties and Delivery of LNs

### Particle Size

**Fig. 2:**
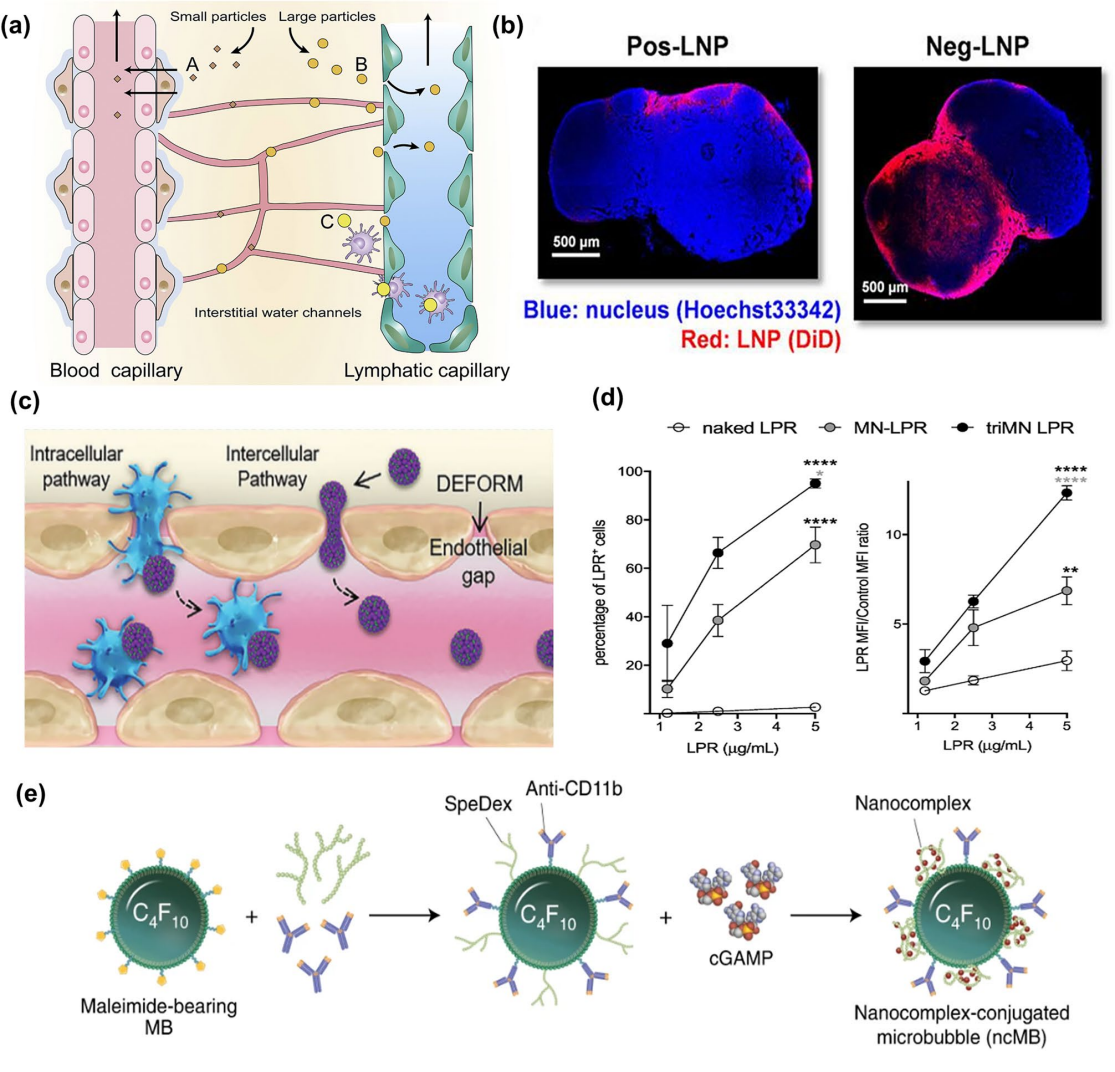
Factors affecting LNs accumulation of nanoparticles. **a** Schematic representation of the entry of particles of different particle sizes into the LNs [42]. Copyright 2017 Elsevier. **b** CLSM images of LNs treated with LNPs with positive and negative charges. LNPs with negative charges infiltrated into the inner area of the LNs [68]. Copyright 2020 American Chem. Society. **c** Schematic illustration of the deformable strategy of lymph-node transfer [83]. Copyright 2021 John Wiley and Sons. **d** The percentages (left) and MFI (right) of fluorescein-labeled naked-, mono-mannosylated- (MN), and tri-mannosylated (triMN) LPR formulations incubated with DC 2.4 cells. The triMN-LPR were significantly better associated with DC 2.4 cells than MN-LPR [93]. Copyright 2018 Elsevier. **e** Schematic illustration of nanocomplex-decorated microbubbles targeting CD11b on APCs [94]. Copyright 2022 Springer Nature

LNs act as a molecular sieve for nanoparticles of various sizes. The particle size is a key factor in determining whether nanoparticles could cross different barriers to enter LNs [42] (Fig. 2a). Small molecules (< 20 KD) or nanoparticles (< 10 nm) can easily enter the capillaries and be rapidly cleared after injection [65, 66]. Although sufficiently small particle size ensures access to capillary lymphatics, the flow velocity of capillaries is 100–500 times higher than that of capillary lymphatics [29], which results in the majority of nanoparticles being cleared in capillaries and few nanoparticles can enter LNs through capillary lymphatics [67]. Large molecules (> 20 kDa) or nanoparticles (10–100 nm) can passively diffuse through the interendothelial junctions into the lymphatic vessel. It has been shown that the optimal particle size range that can be directly transported to LNs based on size effects is 20–50 nm [68–70], which is the basis for the design of targeted nano-delivery systems for LNs. Xiang Yu et al. [71] with the help of the property of suitable size, prepared a lipid nanoparticle α-Melittin-NPs encapsulated with bee venom peptides. Transmission electron microscopy showed that α-Melittin-NPs are spherical nanoparticles with a size of below 30 nm, which can be effectively diverted to capillary lymphatic vessels and LNs, thus providing Melittin works. However, nanoparticles are subsequently restricted to not deep into the LNs because the conduits only allow the entry of small molecules with molecular weights less than 70 kDa or very small particles (2–5 nm) [61, 72]. Therefore, the delivery of goods carried by nanoparticles and retention of T cells, B cells, and other sub-regions in the LNs to enhance humoral immunity is also a research direction worthy of attention.

With the increase in particle size, the efficiency of nanoparticles entering lymphatic vessels decreases. Nanoparticles with a particle size greater than 100 nm are more likely to be trapped in the interstitium, requiring APCs uptake from the interstitium and then entering the LNs [72, 73]. In one experiment, 500 nm fluoresces-labeled nanoparticles were injected into mice with DCs depletion, and no fluorescence was detected in PLNs 48 h later, while fluorescence was detected in the PLNs of mice without DCs depletion, indicating that large particles were transported to LNs in a DCs dependent way [73]. In another experiment, the team of Wang [74] fabricated different sizes of nano-formulations (500, 200 and 50 nm) derived from the yeast cell wall (YCW NPs). Their experiments showed that although all three sizes of YCW NPs could migrate to tumor draining lymph nodes (TDLNs), the accumulation efficiency of YCW NPs on TDLNs was negatively correlated with the size of YCW NPs.

### Charge

The charge can significantly affect the uptake of nanoparticles by APCs. Due to the natural negative charge of the cell membrane, positively charged nanoparticles would exhibit better uptake on APCs compared to the neutral and negatively charged nanoparticles [70, 75]. For example, Zeng et al. [76] exploited the interaction of opposite surface charges to deliver antigenic peptides using positively charged micellar PSA, which significantly improved the uptake of DCs. Although positively charged nanoparticles could significantly enhance the uptake of the APCs, they are more likely to be trapped in the interstitial extracellular matrix composed of collagen fibers and hyaluronic acid after interstitial injection [42], which prevented efficient lymphatic vascular delivery and reduced the accumulation of LNs [77]. In contrast, negatively charged nanoparticles are beneficial to the stealth of immune cells, and are more likely to achieve LNs accumulation through lymphatic vessels [66]. For the LNs accumulation, negatively charged nanoparticles exhibit better accumulation compared to neutral and positively charged nanoparticles [78]. Nakamura et al. [68] prepared neutral, positively, and negatively charged 30 nm liposomes using microfluidic mixing, respectively. The results showed that negatively charged liposomes had the best LNs delivery and were able to penetrate deeper into the inner regions of LNs internalized by DCs most efficiently compared to other charged liposomes (Fig. 2b). In addition, polyethylene glycol (PEG)-modified nanoparticles help to evade macrophage uptake [42, 79]. PEG-functionalized nanoparticles evade rapid clearance by macrophages by reducing protein adsorption, and polyglycerol (PG) has a stronger ability to avoid protein corona formation than PEG [80]. Another study showed that denser PEGylation favors vehicle delivery to LNs [81].

### Deformability

Immune cells such as APCs can cross the endothelial gap (100 nm) despite their large size, mainly due to the soft deformability of their surface [82]. Mimicking the deformability of APCs cells to construct deformable nanoparticles may be helpful to facilitate LNs transport [65]. Based on this, researchers further investigated the effect of the deformability of nanoparticles on the LNs distribution. Song et al. [83] constructed an albumin-stabilized emulsion and demonstrated that the softness and adaptive deformability would be helpful for better LNs accumulation compared to solid particles (Fig. 2c). Moreover, deformability also affects the efficiency of uptake and internalization of cells, even going through different internalization pathways to regulate uptake [84]. In the uptake of nanoparticles by APCs, the uptake of soft nanoparticles was lower than that of hard nanoparticles, possibly due to the short contact time between soft nanoparticles and APCs. A study showed that stiffer discoidal polymeric nanoconstructs (DPNs) exhibited a threefold greater macrophage internalization than soft particles [85]. Therefore, consideration of deformability in the design of nanosystems is a powerful means to overcome the different size barriers in the delivery of LNs.

Different shapes of nanoparticles also affect the accumulation of LNs. Mueller et al. [86] prepared conventional spherical and rod-shaped PRINT hydrogel particles with both sizes less than 100 nm. The experiments showed that rod-shaped nanoparticles could be trafficked more to PLNs and showed time-dependent accumulation within 48 h, while spherical nanoparticles mostly stayed at the injection site. Therefore, changing the shape of nanoparticles is also a strategy to improve the distribution of nanoparticles.

### Active Targeting

The reason why LNs are a key target for tumor vaccination is that the evaluation of vaccine efficacy depends on its ability to enhance adaptive immunity. It is particularly important that the APCs internalized by the vaccine migrate rapidly to reach the LNs and activate various immune cells in the LNs. In this process, the efficiency of vaccine antigen translocation to the interior of APCs is the main rate-limiting step [87]. Therefore, the modification of ligands on nanocarriers to target APCs is a means to promote the immune activation ability of LNs targeted to vaccines. Using DEC-205, Clec9A [88], mannose receptor (MR) [89], DC-SIGN [90], Dectin-1 [91], and Siglec-H [92] receptors, etc. expressed on the surface of APCs and selecting the corresponding ligands for modification is a powerful means to achieve precise APCs targeting and LNs delivery. LE Moignic et al. [93] used an attractive Lipid-Polymer-RNA lipopolyplexes delivery system (LPR) with α-D-mannopyranoside to achieve accurate delivery of mRNA to DCs. Naked, monomannosylated (MN), and tri-mannosylated (triMN) LPR was tested for its ability to bind to DCs. The fluorescence intensity of triMN-LPR was threefold and sevenfold higher than that of MN-LPR and naked LPR, respectively. (Fig. 2d).

Besides, targeted delivery of APCs can be achieved using antibodies such as CD11b and CD11c. For example, Li et al. [94] developed microbubbles modified with CD11b antibodies and delivered 2′3′-cyclic guanosine monophosphate-adenosine monophosphate (cGAMP) loaded by microbubbles into the cytoplasm of APCs by ultrasound (Fig. 2e). It led to the activation of the cGAS-STING pathway, thereby enhancing the immune response. Extensive lymphoid tissue remodeling often occurs in tumors, and abnormal chemokines become beneficial micro-environmental features of LNs delivery [33, 95], which can also guide the targeted distribution of nanoparticles. Balachandran et al. [96] designed an integrated microfluidic chip to construct ZIF-8 MOFs, while its surface was modified with anti-CCL21 DNA aptamer modifications, which enabled a significant enhancement of LNs accumulation.

In addition to LNs targeting by actively targeting APCs, there are also cases of targeting internal structures of LNs to facilitate infiltration. For example, tannic acid (TA), which is abundant in LN conduits, exhibits a high affinity for elastin fibers. Liu et al. [97] prepared TA-modified nanoparticles to directly mediate nanovaccine into the LNs conduits through the lymph node epidermal cells (LECs) gap. Experiments show that TA leads to the deep and long-term retention of antigens and adjuvant in LNs. Furthermore, PNAd mediates lymphocyte recruitment as described above, and PNAd is highly expressed in HEVs in LNs. There have been studies of LNs targeting generated by monoclonal antibodies against PNAs [98]. More interestingly, it is also a strategy to create targets in LNs in advance and reuse them to achieve LNs targeting of nanocarriers [99].

Active transport also can have an impact on LNs reflux. For example, albumin can bind target molecules to lymphatics and draining lymph nodes [100]. Similarly, the lymphatic homing action of lymphocytes can be used to shuttle nanoparticles to LNs [101]. In oral administration, highly lipophilic carriers can bind to lymph lipoproteins in the enterocyte and thus target the intestinal lymphatics [55].

As mentioned earlier, particle size, charge, deformability and active targeting are the preconditions for the design of LNs targeted nano-delivery carriers. The effects of various factors should be considered comprehensively when designing the delivery carriers of LNs.

## Application of Nano-Delivery Systems in LNs

**Fig. 3:**
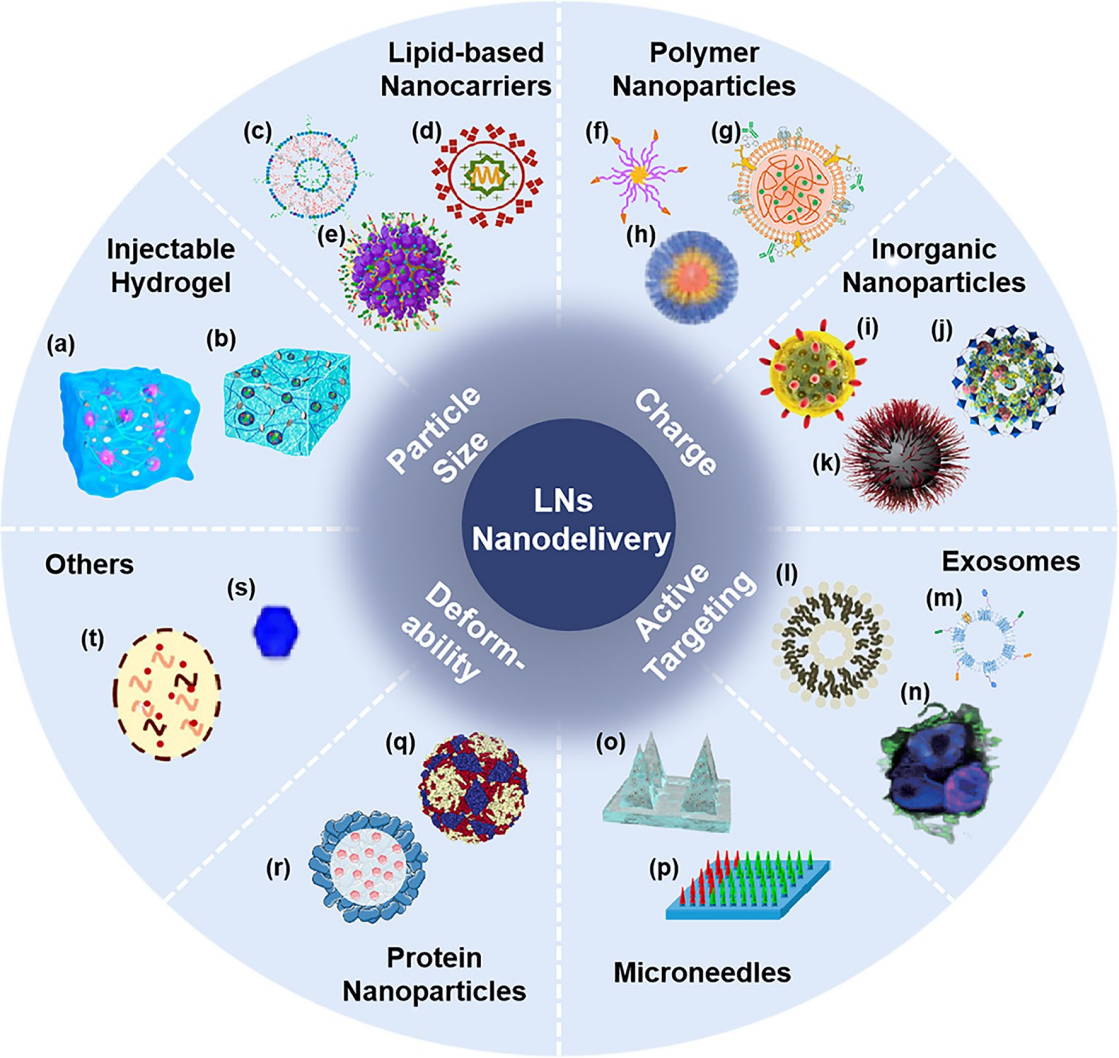
Summary of nano-delivery systems in LNs for tumor immunotherapy. **a** [102], Copyright 2018 American Chemical Society; **b** [103], Copyright 2021 American Chemical Society. **c** [104], Copyright 2019 American Chemical Society; **d** [93], Copyright 2018 Elsevier; **e** [105], Copyright 2022 Elsevier. **f** [106], Copyright 2018 Elsevier; **g** [107], Copyright 2021 American Chemical Society; **h** [108], Copyright 2022 Taylor & Francis Journal. **i** [109], Copyright 2021 John Wiley and Sons;** j** [110], Copyright 2019 Elsevier; k [111], Copyright 2021 Multidisciplinary Digital Publishing Institute. **l** [112], Copyright 2019 John Wiley and Sons; **m** [113], Copyright 2022 Springer Nature; **n** [114], Copyright 2021 The American Association for the Advancement of Science. **o** [115], Copyright 2021 Springer Nature; **p** [116], Copyright 2021 The Proceedings of the National Academy of Sciences. **q** [117], Copyright 2019 Elsevier; **r** [64], Copyright 2018 Elsevier. **s** [118], Copyright 2019 Springer Nature; **t** [119], Copyright 2020 Royal Society of Chemistry

Nano-delivery systems have shown outstanding advantages in the delivery of LNs. Different nanocarriers have unique properties and functions that enable the effective accumulation and immunomodulation of LNs. Here, we summarize eight nano-delivery systems, which each have different advantages in promoting delivery to LNs (Fig. 3).

### Injectable Hydrogel

Hydrogel is a 3D polymer network containing a large amount of water. It has been widely used in biomedicine due to its easy-to-regulate pore size, easy-to-modify surface, and good biocompatibility [120–122]. Hydrogels have long-term retention properties after injection [121], providing superior delivery platforms for the continuous release of immune functional compounds in vivo. Accumulated studies have prepared hydrogel-based vaccines by loading adjuvants and antigens together in hydrogels, so that the antigens can stimulate the immune response in vivo for a long time. Although hydrogel vaccine may not directly involve LNs targeting, the hydrogel could recruit and activate the APCs to promote the APCs migration, thus increasing the accumulation of vaccine components being diverted to LNs [26]. This ability to facilitate sustained vaccine delivery and LNs localization has been studied by researchers. Wang et al. [123] found that compared with vaccines based on single and multiple injections, using hydrogel as the vaccine repository extended the retention window of the injection-site vaccine, accompanied by rapid and sustained recruitment of APCs. Abundant vaccine accumulation was shown in the whole paracortex of the LNs, especially in the T cell area, showing the best antitumor efficacy. Song et al. [124] designed a 3D porous hydrogel assembled from synthetic PEGylated polypeptide copolymers loaded with antigen and TLR3 agonists. It can effectively promote DCs maturation, improve antigen retention and promote antigen drainage to LNs through "long-lasting effect".

**Fig. 4:**
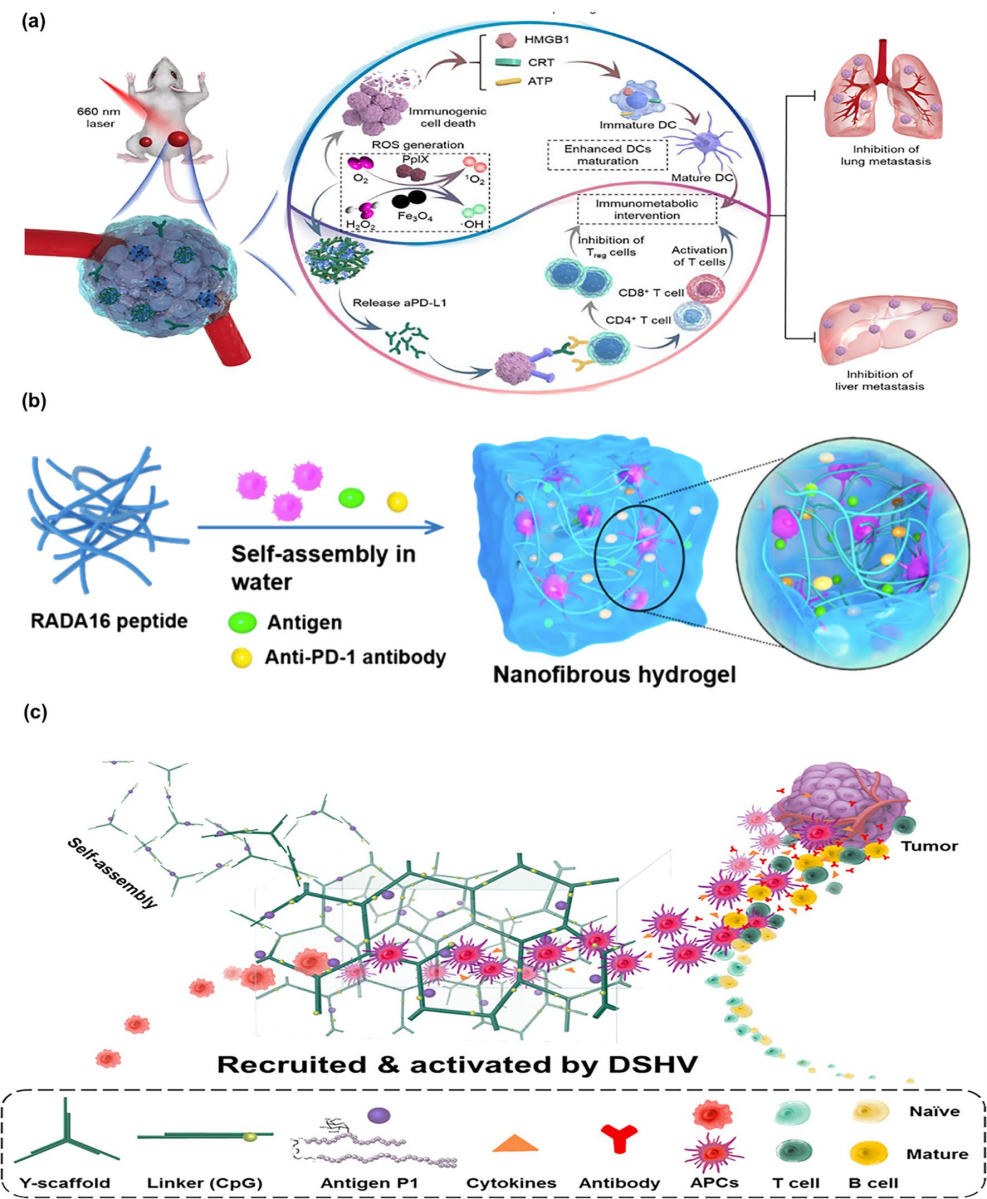
**a** Prodrug hydrogel-mediated PDT/CDT/immunotherapy to treat primary and distant tumors and prevent metastasis [125]. Copyright 2022 Elsevier. **b** Schematic diagram of the structure of peptide hydrogel [102]. Copyright 2018 American Chemical Society. **c** Schematic illustration of the recruitment and activation of host APCs by DNA hydrogel [128]. Copyright 2018 American Chemical Society

Replacement of specific antigens with other substances, such as certain chemotherapeutic agents (anthracyclines, mitoxantrone, oxaliplatin, paclitaxel, cyclophosphamide, bortezomib), photosensitizers in photothermal and photodynamic therapy or radiotherapy sensitizers for radiotherapy, can induce immunogenic cell death (ICD). The occurrence of ICD in tumor cells can release damage-associated molecular patterns (DAMPs) and tumor antigens, which produce individual-specific maturation of APCs and initiation of immune responses. For example, Ding et al. [125] designed a tumor microenvironment and near-infrared light (NIR) -responsive hydrogel using alginate. The accumulated ROS and OH^−^ of PDT under NIR irradiation amplified the ICD effect and disrupted the ROS response junction to achieve the on-demand release of drugs, which significantly promoted the maturity of DCs in the tumor drainage LNs (Fig. 4a). In addition, chemokines recruiting APCs were co-loaded into the hydrogel on the basis of antigen encapsulated adjuvants, such as GM-CSF [126], and CCL21 [127]. Chemokines can promote the enrichment of APCs and significantly enhance the antigen presentation effect of APCs and the efficacy of immunotherapy.

Hydrogels come in a variety of compositions, such as peptides, DNA, polymers, and certain inorganic materials (Table 1). The special fibrous structure of peptide hydrogel scaffolds can mimic the extracellular matrix for 3D cell culture, which supports the delivery of viable exogenous APCs. For example, Yang et al. [102] used peptides to build hydrogel to load anti-PD-1 antibody and antigen (Fig. 4b). LNs were recovered after subcutaneous injection and demonstrated that the peptide hydrogel cell carrier increased the migration of exogenous DCs to LNs and recruited a large number of autologous DCs to promote T-cell immunity. The direct use of DNA molecules to build hydrogels has good biocompatibility, sequence programmability, and tunable versatility. By using X-, T- or Y-shaped DNA structural units as hydrogel scaffolds, rapid DNA hydrogel preparation can be achieved, which is an effective means of activating immunity. Shao et al. [128] developed a DNA hydrogel (DSHV), consisting of a Y-type scaffold and a linker containing a CpG sequence. It was able to effectively activate APCs and induce a strong immune response after loading antigens (Fig. 4c). The construction of biocompatible hydrogels based on polymeric materials is the most common type of gels at present, and gelatin, F127, chitosan, sodium alginate, and hyaluronic acid have been widely used to construct hydrogels to enhance immune therapeutic efficacy. A study combined sodium alginate (ALG), ATP-specific aptamers (Aapt, DNA sequences that bind specifically to ATP and form tertiary structures), and CpG oligonucleotides (CpG ODNs, extended strands complementary to Aapt) into a smart hydrogel. It showed ATP-triggered CpG release from radiotherapy, which significantly enhanced APCs activation compare with the ability of radiotherapy alone [129]. In addition, the use of rigid inorganic materials to build hydrogel scaffolds is an emerging approach. For example, injectable vaccines formed by coupling mesoporous silica microrods (MSRs) with mesoporous silica nanoparticles (MSNs) can form three-dimensional macroporous scaffolds after injection. Scaffolds recruited DCs into the scaffold to achieve effective maturation under the co-stimulation of antigen and adjuvant [130].

**Table 1:** Summary of injectable hydrogels for LNs immunotherapy

Type of injectable hydrogel	Carrier material	Loading drug	Type of cancer	References
Peptide hydrogel	Epitope-peptide conjugate	OVA epitope	E.G7-OVA tumor model	[131]
Peptide hydrogel	OVA epitope SIINFEKL	OVA epitope SIINFEKL	/	[132]
Peptide hydrogel	PEG-b-poly(L-alanine)	Tumor cell lysates, GM-CSF, anti-CTLA-4/PD-1 antibody	B16 tumor model	[133]
DNA hydrogel	Poly CpG DNA	Poly CpG DNA, c-di-GMP, and melanin	CT26 colon tumor model	[134]
DNA hydrogel	CpG DNA	Ethylenediamine-conjugated OVA	EG7-OVA tumor model	[135]
Polymeric hydrogel	Polymers gelatin and Pluronic® F127	S-nitrosoglutathione and antagonizing CTLA-4 mAb	B16F10-OVA tumor model and 4T1 tumor model	[136]
Polymeric hydrogel	Oligo (ethylene glycol) methacrylate (OEGMA)	OVA and R837	B16-OVA tumor model	[103]
Polymeric hydrogel	HA-PCLA	GM-CSF, pOVA polyplex	B16F10-OVA Lung Metastasis tumor model	[137]
Polymeric hydrogel	PDLLA-PEG-PDLLA	ICG, R848, and CpG ODN	4T1-Luc tumor model	[138]
Inorganic nanocomposite hydrogel	Alginate and graphene oxide	OVA, GM-CSF, and CpG	B16-OVA tumor mode	[139]
Inorganic nanocomposite hydrogel	Graphene oxide and poly-ethylenimine	OVA-mRNA and R848	B16-OVA tumor model	[140]

At present, injectable hydrogels show wide application prospects in tumor immunotherapy. Researchers continue to combine hydrogel-based immunotherapy with other therapy, and develop hydrogels with functional diversity to improve treatment outcomes.

### Lipid-Based Nanocarriers

Lipid nanoparticles (LNPs) have been occupying an important position in the field of drug delivery, which exhibit advantages such as the simple structure, ease of modification, high bioavailability, biodegradability, low toxicity and safety [141]. Lymphatic vessels are channels for transporting lipophilic substances [142], and the lipophilic nature of liposomes makes them susceptible to phagocytosis by macrophages of the reticuloendothelial system, thus achieving natural enrichment of LNs sites [143]. Therefore, lipid-based nanocarriers are a class of drug carriers of interest in lymphatic-targeted drug delivery systems.

**Fig. 5:**
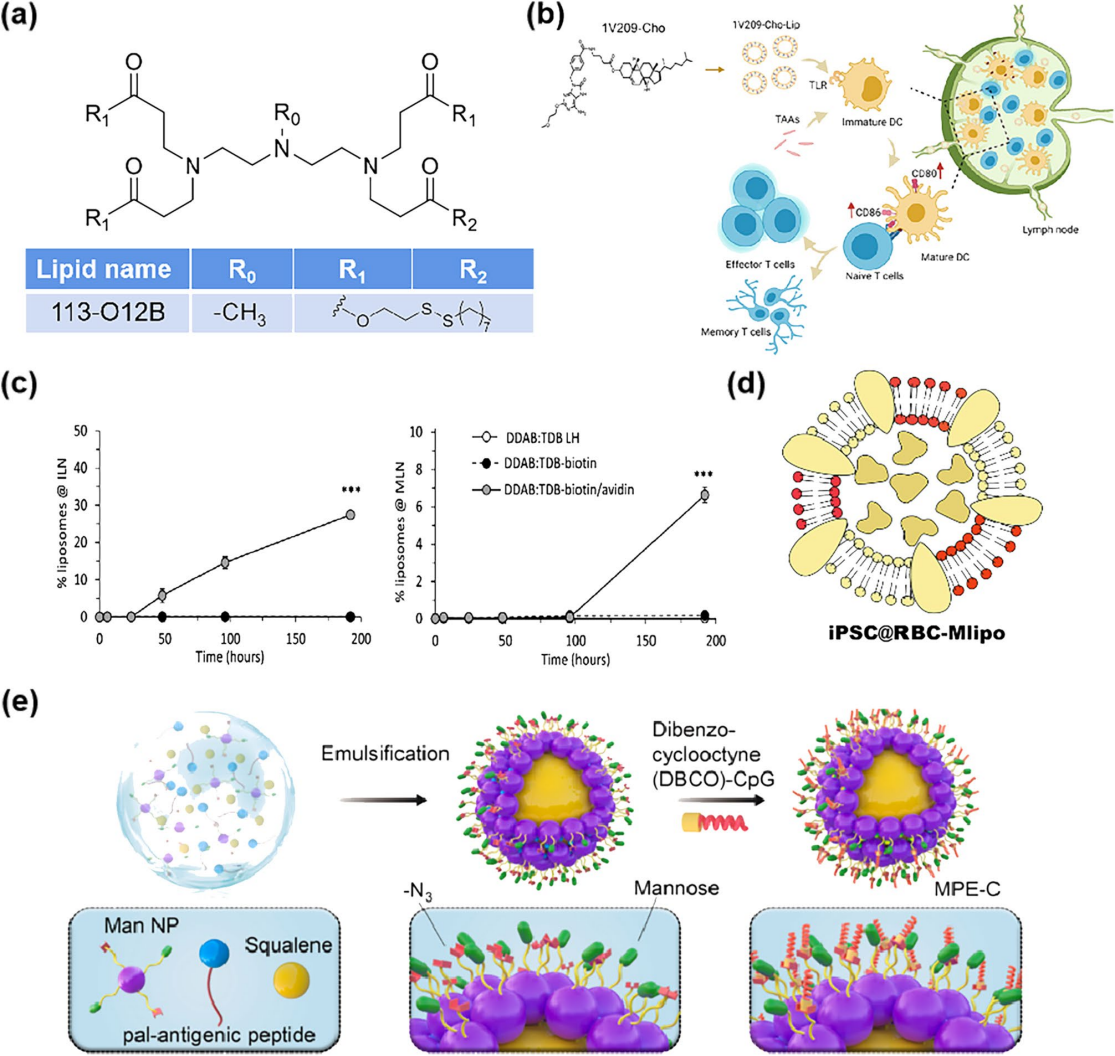
**a** Schematic illustration of the top lipid (113-O12B) for mRNA delivery to LNs after screening in the library of the team of XU Q [144]. Copyright 2022 The Proceedings of the National Academy of Sciences. **b** Schematic Illustration of the cholesterolized TLR7 Agonist Liposomes for Eliciting Immunity [151]. Copyright 2021 American Chemical Society. **c** Percentage of biotin-DDAB: TDB liposomes detected at the inguinal lymph nodes (ILNs, left) and mesenteric lymph nodes (MLNs, right). When mice received a predose of avidin were the DDAB: TDB-biotin liposomes more retained at the ILNs and MLNs [104]. Copyright 2019 American Chemical Society. **d** Schematic of iPSC@RBC-Mlipo by fusing erythrocyte membrane (membrane in red) with M-liposome (membrane in yellow) and packaging iPSC protein [154]. Copyright 2021 The American Association for the Advancement of Science. **e** Schematic illustration describing the construction of mannosylated Pickering emulsion loaded with CpG and pal-antigenic peptide (MPE-C) [105]. Copyright 2022 Elsevier

The lymphatic targeting and retention ability of liposomes or LNPs can be further optimized by special design. In addition to improving physical properties such as size and charge as described above, enhancing the targeting ability by changing the formulation of liposomes is a promising strategy to achieve LNs accumulation. The team of Xu [144] suggested that changing the head chemistry of liposomes can affect their delivery and targeting. In their latest study, 113-O12B lipid molecules were screened out from the structure library, which can target LNs and liver respectively in a ratio of 3:1 (Fig. 5a). The team demonstrated that these 113-O12B endogenous liposomes can deliver more mRNA to APCs compared to the lipid molecule ALC-0315 used in the Pfizer/BioNTech mRNA vaccine. Furthermore, studies have shown that lymphatic vessels have a higher clearance rate of liposomes containing phosphatidylserine (PS), which may lead to preferential uptake by macrophages [142, 145]. According to this view, the team of Luozhong [146] combined a bionic strategy to introduce PS into the formulation of LNPs to deliver mRNA. PS binds to cell surface scavenger receptors and acts as a "eat me" signal to phagocytes to increase their endocytic activity, leading to effective protein expression in LNs and spleens. More interestingly, Li et al. [147] proposed menthol liposomes (Men-nanoLips) for increasing the accumulation of LNs in DC vaccines using the permeability-enhancing properties of the traditional Chinese medicine menthol. They also disclosed the immunological mechanism of menthol enhanced LNs targeting.

Recent studies have revealed a close relationship between cholesterol transport and lymphatic transport [148, 149]. Lim et al. [150] showed that lymphatic transport of cholesterol can not only passively enter the lymphatic vessels into LNs, but also can rely on scavenger receptor type B class I (SR-BI) expressed by lymphatic endothelial cells to participate in LNs transport. Exploiting the nature of lymphatic transport of cholesterol to achieve effective LNs delivery of drugs is an efficient means. Wan et al. [151] developed a liposome of TLR7 agonist 1V209 and cholesterol coupling (Fig. 5b). Compared to liposomes loaded with 1V209, the 1V209-Chol coupled liposomes achieved 7.35-fold and 2.06-fold enhanced accumulation of LNs at 12 and 24 h after administration. Therefore, the design of LNs delivery nanocarriers with adequate consideration of the role of cholesterol in enhancing LNs accumulation is an emerging and effective strategy in recent years.

In the latest research, the strategy to achieve active targeting has expanded beyond simple modification with ligands or antibodies. Rather, this extends to the use of a substance pre-positioned in vivo with a "specific binder" carried by a later input nanoparticle to accomplish a simulated "ligand-receptor" interaction. A more typical pair of affinity complexes are avidin and biotin, Roces et al. [104] prepared a liposomal vaccine adjuvant based on microfluidic production technology. Biotin was distributed on the surface, which could bind to the pre-injected affinities to form high-affinity complexes, thus improving the liposome accumulation and retention capacity in LNs (Fig. 5c). Of course, such "ligand-receptor" interactions showed great potential for development and design specific chemical reactions between the two substances are introduced. Interestingly, Qin et al. [99] innovatively used click chemistry between azide and dibenzocyclooctyne (DBCO) moieties. First, their group injected a polymer containing azide, 24 h later, liposomes cocarrying DBCO modified antigen and adjuvant were injected. DBCO could react with the azide group, resulting in the accumulation of antigen and adjuvant in LNs.

The phospholipid bilayer structure of liposomes is similar to the cell membrane composition. The fusion itself is the process by which the drug carried by the liposome enters the cell [152, 153]. This allows liposomes to fuse easily with the plasma membrane of other cells to form bionic liposomes, thus naturally inheriting the unique functions from other biological membranes. iPSC@RBC-Mlipo, an erythrocyte membrane fusion liposome nanovaccine, was prepared by Zhai (Fig. 5d) [154]. They argued that damaged erythrocytes can achieve splenic targeting because of interception and retention in flow through the spleen. The experiments showed that fused liposomes can fully accumulate in the spleen and has a remarkable ability to promote the recruitment, maturation, and antigen cross-expression of APCs.

In addition, lipid-based nanocarriers used for targeting LNs also include lipid emulsions, which can improve the efficiency of LNs targeting by improving the softness of the nanovaccine. MA G’s group [105] is dedicated to the study of using Pickering emulsions to enhance vaccine response. In the group's latest results, the high specific surface area and soft deformability of Pickering emulsions enhanced the interaction and uptake with mannose receptors of APCs, thus allowing the vaccine to be taken into LNs to exert powerful anti-tumor immunity (Fig. 5e).

In summary, lipid-based nanocarriers are widely used in LNs delivery and are one of the fastest developing carriers. Researchers continue to use novel strategies to expand the possibility of liposome targeting. At the same time, liposomes are the most commercially available nanocarriers in nanomedicine. lipid-based nanocarriers are considered as the most promising LNs delivery systems.

### Polymeric Nanoparticles

Polymeric nanoparticles have excellent drug loading capacity and controlled drug release behavior, and can improve the targeted distribution of drugs [155]. Polymeric nanoparticles have structural diversity and their surface can be modified or engineered accordingly to the desired properties [156]. These properties allow researchers to select ideal materials and incorporate novel design ideas such as specific degradation, conversion, and response mechanisms to improve targeting and retention during the delivery of LNs. Therefore, polymeric nanoparticles can be an attractive option for targeted drug delivery carriers for LNs (Table 2).

**Table 2:** Summary of polymeric carrier Mater for LNs immunotherapy

Polymeric carrier material	Loading drug	Type of cancer	References
PPS	CpG	EL4 tumor model	[165]
PLGA	TLR 7/8 agonists	B16F10-OVA tumor model, MB49 tumor model and Renca-GL tumor model	[166]
PLGA	Imiquimod	B16-OVA tumor model, B16F10-Luc tumor model and CT26 tumor model	[107]
PEG-PE	MPLA and OVA/E7	TC-1 tumor model	[164]
PEG-b-PAsp	TRP2 peptide	B16F10 tumor model	[108]
PLA-PEI	CpG and OVA	B16-OVA tumor model, MC38 tumor model and E0771 tumor model	[158]
DSPE-PEG2000	IR-780	4T1 tumor model	[167]
DSPE-PEG	PTX	4T1 tumor model and 4T1-luc tumor model	[106]
Ct	OVA	B16F10-OVA tumor model	[168]
MPDA	R837	B16-OVA tumor model	[159]

**Fig. 6:**
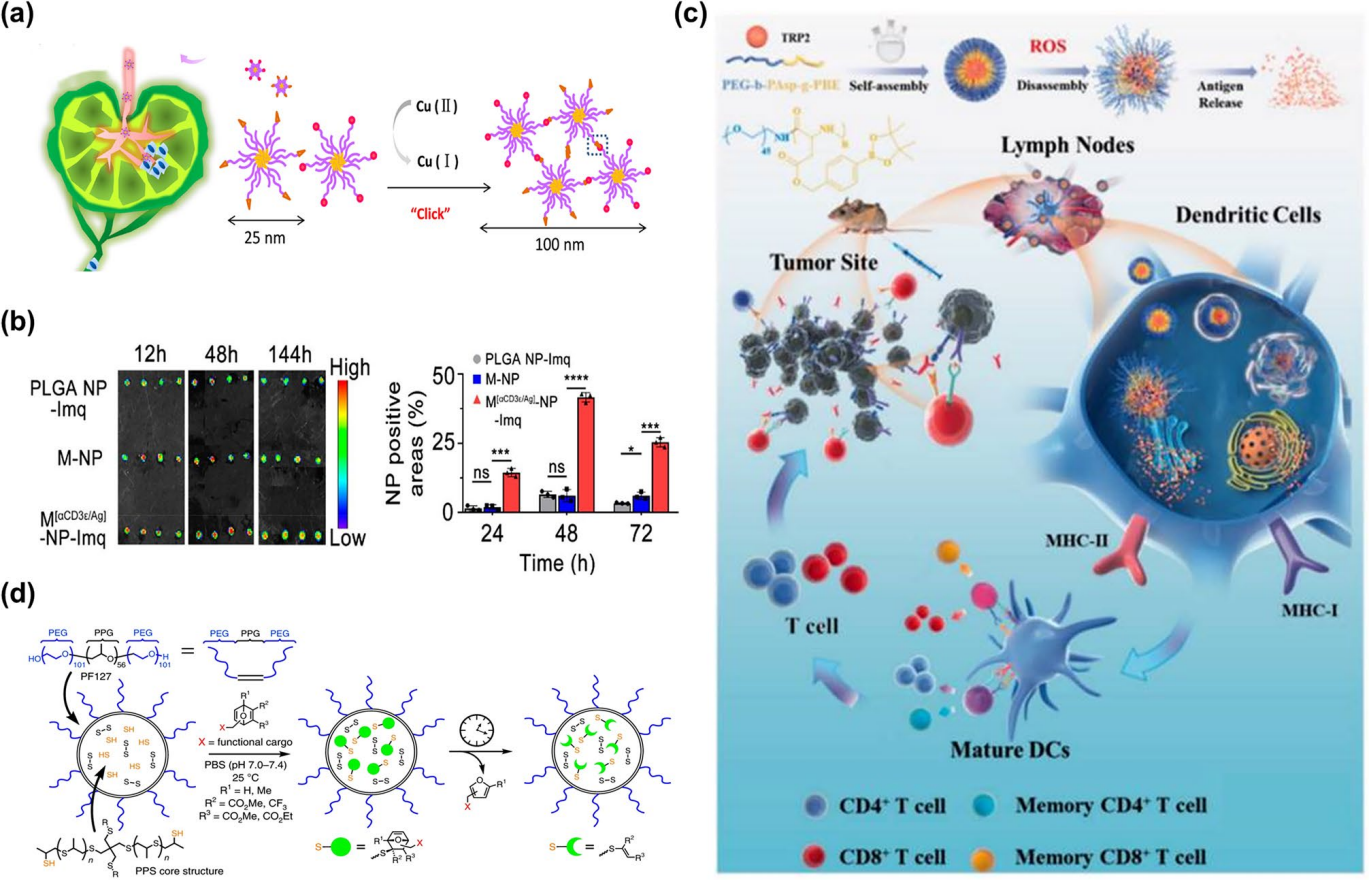
**a** Schematic illustration of DSPE-PEG micelles accumulating in LNs [106]. Copyright 2018 Elsevier. **b** Ex vivo fluorescence imaging of excised LNs (left) and percentage of DiI positive areas in the field of LN sections after treatment (right). Nanoscale aAPCs are efficiently accumulated and retained in LNs [107]. Copyright 2021 American Chemical Society. **c** Schematic illustration of PEG-b-PAsp-g-PBE/TRP2 as a nanovaccine delivery system [108]. Copyright 2022 Taylor & Francis Journal. **d** Schematic representation of PPS NP preparation, conjugation with OND electrophiles, and Retro-Diels–Alder release of furan-tagged cargo [165]. Copyright 2020 Springer Nature

To ensure optimal accumulation of LNs, an equally appropriate particle size is necessary to achieve passive targeting. However, fixed size NPs cannot achieve both effective "penetration" and long-term "retention". So Qin’s team [106] designed a polymeric micelle with size conversion and azide/alkyne groups on the surface of DSPE-PEG micelles for click chemistry. Initially, small micelles of about 25 nm can effectively enter the LNs to treat the metastases of the tumor. After reaching the primary tumor, a cycloaddition reaction occurs between the groups on the micelle, resulting in the formation of micelle aggregates of about 120 nm (Fig. 6a). As the exocytosis of nanoparticles is reduced and the reflux into the blood is minimized, the drug has the best antitumor effect.

Negatively charged nanoparticles exhibited better lymphatic accumulation compared to neutral and positively charged nanoparticles, Nishimoto et al. [157] studied this phenomenon in more detail. They compared the correlation between three anionic dendritic polymers containing different terminal groups. This experiment suggested that the efficiency of LNs targeted drug delivery can also be improved by controlling the terminal structure of anionic polymers, and provided an idea for the design of LNs targeting polymers. In addition to the selectivity of lymphoid tissue for nanoparticle charge, electrostatic adsorption due to the charge provides some convenience for nanoparticle drug loading. Xu et al. [158] reported a mannanosan-modified pathogen-like polymeric nanoparticle as a protein vaccine carrier. And using cationic nanoparticles assembled by polylactic acid-polyethylenimine (PLA-PEI) as the core to attract antigenic proteins and CpG to the nanoparticle by electrostatic interaction. The mannan modification significantly enhanced the LNs drainage ability of the nanovaccine and promoted the capture of DCs in the LNs.

Polymeric nanoparticles can be used in a wide range of materials. Special chemical groups on the surface make it possible for the rational design of functionalization. Through surface modification, some disadvantages of nanoparticles can be avoided in the delivery process. For example, one study conducted by WANG L loaded the TLR-7 agonist imiquimod (R837) into mesoporous polydopamine (MPDA) nanoparticles. To ensure smooth lymphatic drainage, polyvinylpyrrolidone (PVP) was surfacing coated on the MPDA. This change in surface properties enhanced the LNs penetration and retention ability of nanoparticles. And it remained in the SCS and interfollicular region 24 h after injection, increasing the chance of DCs meeting T cells [159]. Instead of improving lymphatic exposure through chemical modifications, biomimetic nanotechnology provides a simple strategy for nanoparticles to naturally express a variety of markers required for cellular communication. The development of metabolic glycoengineering also makes it possible to introduce other chemical labels to cell membranes. Based on the development of these two technologies, Xiao et al. [107] cleverly designed artificial antigen-presenting cells (aAPCs). Poly (lactate-co-glycolic acid) (PLGA) nanoparticles were coated with DC film modified with CD3ε antibody (αCD3ε) by click-chemical modification. The fluorescence signal accumulation and retention in the LNs was 7.6 times higher than that in the control group at 24 h after subcutaneous injection (Fig. 6b). The DC membrane retains the natural proportion of MHC I-Ag and CD28 co-stimulators, directly enhance anti-tumor immunity.

In addition, the construction of specific environmental stimulus-responsive polymeric nanoparticles can significantly promote the accumulation of LNs for drugs. In our group [160], novel pH/redox dual-sensitive micelles Trp2/CpG-NPs were prepared using poly(l-histidine)-poly (ethylene glycol) (PLH-PEG) as a skeleton, which can effectively deliver to the LNs due to suitable particle size. At 48 h after subcutaneous injection, the accumulation efficiency of LNs was 8.12-fold higher than that of the control group. Wang et al. [108] constructed TRP2 nanovaccine. The nanovaccine consisted of a phenylboronic ester (PBE)-grafted diblock copolymer (PEG-b-PAsp-g-PBE) loaded with TRP2 peptide tumor antigen, where PBE can be selectively degraded by intracellular ROS triggers (Fig. 6c). They demonstrated that the TRP2 nanovaccine was effectively captured by LNs and increased DCs uptake, relying on increasing negative charge, ROS response, and pH response. More interestingly, Karabin et al. [161] used poly (ethylene glycol)-bl-poly (propylene sulfide) (PEG-bl-PPS), a block copolymer, to make a self-assembled filomicelle (FM) scaffolds. It can degrade from cylindrical shapes to spherical monodisperse micelles with diameters below 40 nm under photo-oxidation or physiological oxidation. Because spherical micelles fall within the optimal range for lymphatic vessel transport after subcutaneous injection, they can efficiently deliver encapsulated drugs from the interstitial space to lymphatic tissue. Furthermore, this FM scaffold supports one month of continuous long-lasting delivery of the contained drug to stimulate APCs, thus enhancing the effect of immunotherapy.

Given the anatomical structure of the LNs, it is critical to enhance the carriers' ability to overcome biological barriers and destabilizing obstacles in vivo to obtain the best therapeutic effect [162]. Liang’s team [163, 164] has shown in two successive studies that PEG-PE-based micelles can rapidly target LNs. The micelles can drain into the central region of LNs co-localizing with the duct network and remaining there for more than 96 h. This team used this carrier for antigen and adjuvant delivery and found that the PEG-PE micelles-based vaccine effectively delivered antigen to CD8αDCs, eliciting more efficient presentation and stronger CD8^+^T cells initiation. Delivery of nanoparticles to LNs, although with greater lymphatic uptake capacity, have difficulty in accessing the lymphocyte compartment within LNs through 3–5 nm conduits, which becomes a barrier to the internal transport of LNs. To address this issue, Alex Schudel et al. [165] developed a two-stage approach to deep LNs delivery of drugs. They used thiol-reactive oxanorbornadiene (OND) linkers to load the drug onto PPS nanoparticles. This platform regulated the timing of half-life drug release according to the first-order Retro-Diels–Alder mechanism, which controlled the release of mobile lymphatic small molecule cargo (Fig. 6d). Delivery to specific lymphocyte subpopulations in the cortex and paracortex of LNs was achieved compared to particles or free compounds alone. Finally, the number of target cells (T and B cells and DCs) was increased by several orders of magnitude.

Polymeric nanoparticles have great innovation potential, in part because the rational design of polymers is in parallel with the development of chemistry. Industrial production and the safety of organic reagents should also be taken into account when creating carriers with special functions.

### Inorganic Nanoparticles

Inorganic materials enable the effective delivery of LNs for vaccines or immune drugs. Inorganic nanoparticles have the following advantages: structurally stable; can be tailored into carriers with specific physical properties (size, porosity, electrical conductivity, hydrophilicity, and optical-magnetic properties, etc.); high control degree; huge specific surface area and excellent surface-selective adsorption capacity; easy surface modification for enhanced targeting ability and enhanced immune system escape; combination of various tumor therapies, showing great promise in combination immunotherapy [169]. Common inorganic nanoparticles used for LNs delivery include mesoporous silica nanoparticles (MSNs), gold nanoparticles (AuNPs), metal–organic backbones (MOFs), and iron oxide nanoparticles (Table 3).

**Table 3:** Summary of Inorganic carrier materials for LNs immunotherapy

Inorganic carrier materials	Loading drug	Type of cancer	References
Mesoporous silica	OVA and CpG	B16-OVA tumor model	[176]
Mesoporous silica	CpG	B16F10-OVA tumor model	[171]
Mesoporous silica	OVA and R848	/	[177]
pSi	RAPA	/	[172]
Au	OVA	/	[111]
Au	pCMV-MART1	B16F10-OVA tumor model	[178]
SPIO	OVA	/	[175]
MOF	OVA and CpG	EG7-OVA tumor model	[110]
MOF	CpG	TUBO tumor model	[179]

**Fig. 7:**
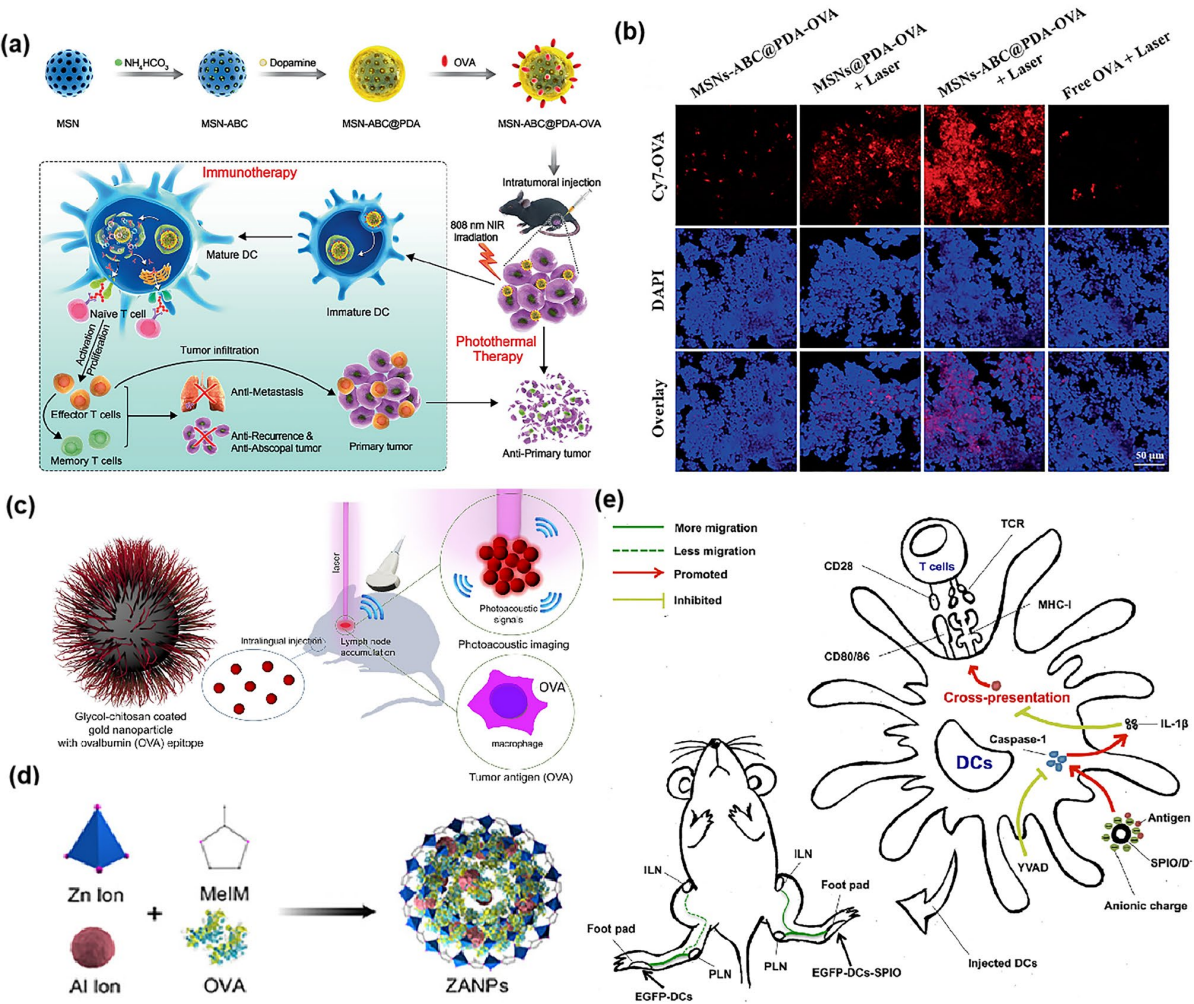
**a** Schematic illustration of nanovaccine preparation and the enhancement of PTT and B16-OVA for melanoma immunotherapy. **b** The confocal fluorescence images of the ex vivo LNs stained by DAPI. ABC and laser irradiation can significantly promote nanovaccine migration to the LNs [109]. Copyright 2021 John Wiley and Sons. **c** Schematic structure of GC-AuNPs and LNs accumulation in photoacoustic (US/PA) imaging [111]. Copyright 2021 Multidisciplinary Digital Publishing Institute. **d** Schematic diagram of the composition and structure of ZANP [110]. Copyright 2019 Elsevier. **e** Graphical abstract of SPIO as a nano-adjuvant for DCs [175]. Copyright 2018 Springer Nature

MSNs surface contains negatively charged and hydrophilic silicol groups (SiOH). The super-porous structure ensures the effective loading of high molecular weight antigens and immune-stimulating molecules, which makes MSNs a potential LNs targeted vaccine carrier. In addition, mesoporous silica material is potential adjuvant that can enhance the immune response through inflammasome activation [170]. Hu et al. [171] prepared a biomimetic nanovaccine by wrapping B16F10 mouse melanoma cell membranes in the outer layer of MSNs using highly automated flash nanocomplexation (FNC). In vivo uptake experiments showed that the surface antigens of cancer cell membranes gave them APCs targeting effects. In recent years, the combination of vaccine delivery and photothermal therapy (PTT) for tumor ablation using MSNs as carriers has also been reported. Huang et al. [109] loaded ammonium bicarbonate (ABC) in MSNs pores, surface coated with the photothermal agent PDA and coupled with thiolated OVA for melanoma PTT immunotherapy. This therapeutic vaccine released OVA under the dual disruption of NIR laser irradiation and NH-induced temperature increase, and the gas generated by ABC disintegration in an acidic environment accelerated the release of antigen and migration to LNs (Fig. 7a, b). In addition to serving as vaccine carriers, MSNs are also used for the delivery of immune drugs. For example, Stead et al. [172] designed porous silicon (pSi) nanoparticles loaded with the immunosuppressant rapamycin and surface coupled with DC-SIGN antibodies targeting DCs.

AuNPs are widely used for imaging LNs due to their strong light absorption and light scattering by localized surface plasmon resonance (LSPR) [173]. Sun et al. [111] developed glycol-chitosan-coated gold nanoparticles (GC-AuNPs) that highlighted LNs in ultrasound-guided photoacoustic (US/PA) imaging (Fig. 7c). LNs 3D analysis showed that the accumulation of GC-AuNPs in LNs increased with time. However, some studies have shown morphological changes in the liver, spleen, and LNs of adult rats during the long-term administration of GNPs [174]. And the accumulated gold nanoparticles induce oxidative stress through ROS production, so their safety remains the biggest obstacle for clinical application.

MOFs are a rapidly developing coordination polymer in the last two decades because of their tunable pore size, high surface area, and high loading capacity. Currently MOFs can also achieve effective LNs delivery of vaccines. For example, Zhong et al. [110] synthesized a zeolitic imidazole backbone (ZANPs) with pH-sensitive degradation properties for the delivery of OVA and GpG (Fig. 7d). Experiments results showed that after subcutaneous injection, ZANPs could target and efficiently accumulate in LNs, and both antigen and adjuvant were efficiently internalized by LNs-resident APCs.

Otherwise, iron oxide nanoparticles have been reported as a LNs target delivery carrier to carry antigens for inducing the migration of DCs to LNs previously (Fig. 7e) [175]. But reports in recent years have mainly focused on LNs tracers for contrast imaging.

In conclusion, inorganic nanomaterials offer obvious advantages for vaccines and immunotherapies and have a wider application as targeting carriers for LNs in tumor immunotherapy. However, a balance between effectiveness and safety must be struck when designing inorganic nanoparticles for nanomedicine, so that inorganic nanomaterials can realize their full clinical potential.

### Exosomes

Since the discovery of exosomes in the supernatant of sheep erythrocytes cultured in vivo in 1983, researchers' knowledge of exosomes has evolved from redundant membrane proteins to the gradual recognition of the important role of exosomes in the body's immune response [180]. Exosomes are 30–100 nm bilayer vesicles actively released by cells [181] and are a type of extracellular vesicles (EVs), which are in the ideal size range for lymphatic transport. In recent years, research related to the use of exosomes as delivery vehicles for LNs has continued to rise.

**Fig. 8:**
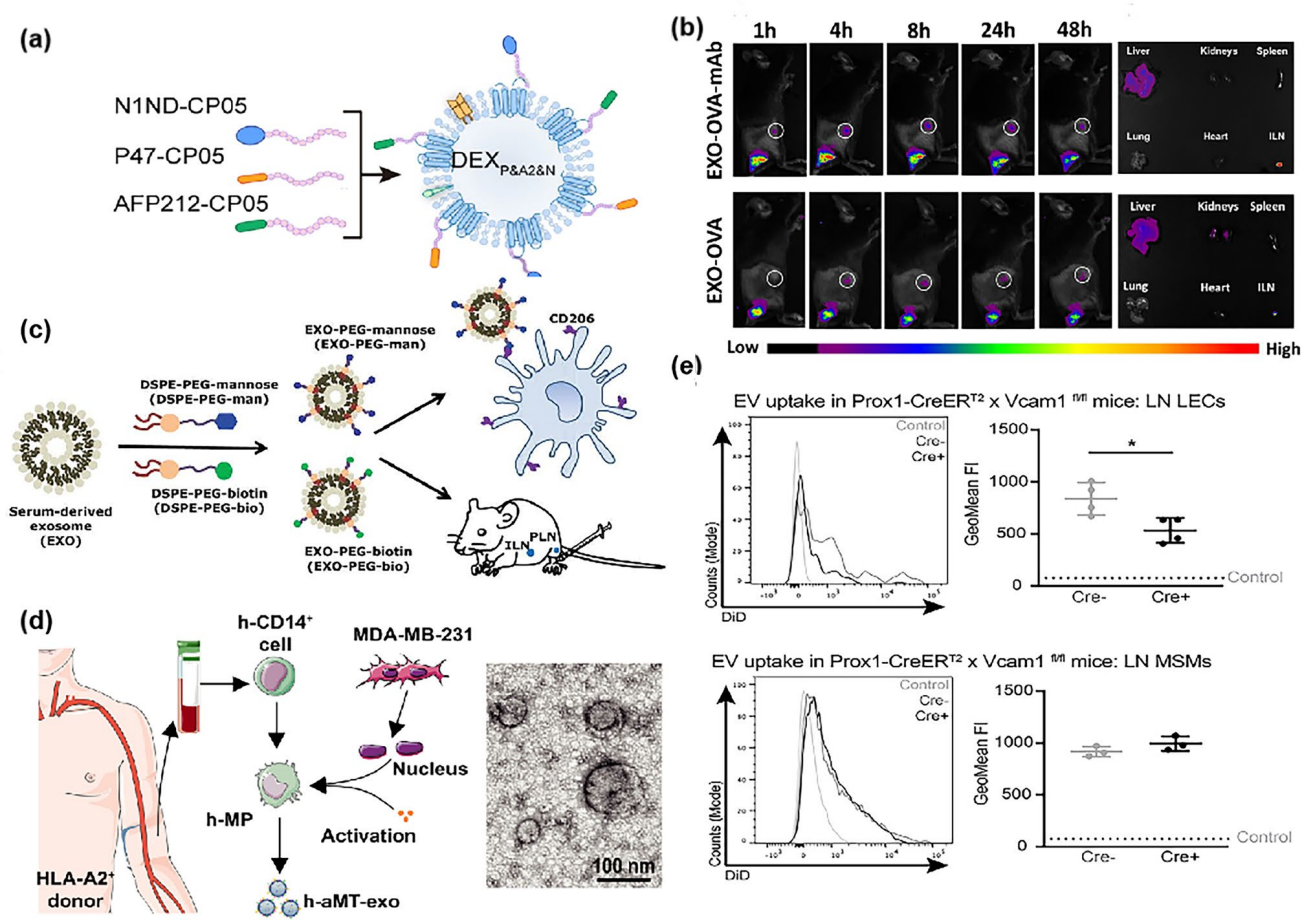
**a** Schematic illustration for designer DEX vaccine-DEX_P&A2&N_ [113]. Copyright 2022 Springer Nature. **b** Fluorescence images of mice and main organs (circle ILNs). Exosomes are more than 150 times stronger in ILNs than in any other tissue [184]. Copyright 2020 Elsevier. **c** Schematic illustration of the fabrication of PEG-EXO-man for targeted delivery into the DCs and LNs [112]. Copyright 2019 John Wiley and Sons. **d** Schematic diagram of the preparation of human PBMC-derived exosomes containing tumor cell nuclei (left) and their TEM images (right) [114]. Copyright 2021 The American Association for the Advancement of Science. **e** B16F10-derived EVs labelled with DiD were injected into tamoxifen-treated Prox1-CreER^T2^ x Vcam1^fl/fl^ mice. Example histograms and quantification of DiD uptake in PLN LECs (above) and MSMs (below). The selectivity of EVs and TDLNs in melanoma cells depends on the expression of lymphoid VCAM-1 [193]. Copyright 2022 John Wiley and Sons

An increasing number of studies have shown that normal immune cell-derived exosomes are involved in a variety of immune processes, such as antigen presentation and immune cell activation and inhibition [182]. Therefore, the use of immune cell exosomes for enhancing immunotherapy is an attractive avenue. Among these, DC-derived exosomes with the homing ability of LNs after activation have been mostly studied. Exosomes no longer stop at "bare" exosomes, but functionalized and modified exosomes have become a hot topic of research. For example, Zuo et al. [113] developed a liver cancer immunotherapeutic vaccine DEX_P&A2&N_ using DCs-derived exosomes coated with a targeted peptide binding to exosomal anchor peptide (CP05), antigenic epitopes, and immune adjuvants (Fig. 8a). Notably, higher fluorescence was detected in the spleen, mesentery, and ILNs of mice after receiving DEX than in the PBS control group. To further enhance the LNs homing ability of DCs-derived exosomes, the researchers functionally engineered DCs exosomes using CD62L for efficient delivery to TDLNs by enhancing the interaction with lymphatic endothelial cells (LECs). Researchers loaded cel-miR-54 in exosomes to validate targeting efficiency, which eventually showed striking results of hundreds of times higher accumulation levels in mouse LNs than in the unmodified group [183]. However, since antigen-specific T cell function is often suppressed, other researchers have combined cancer vaccines and checkpoint blockade strategies to prepare bifunctional exosomes that synergistically enhance antitumor immune responses. The team of Phung [184] combined exosomes with CTLA-4 blockade therapy and reported an OVA pulse-activated DCs-derived exosomes modified by a CTLA-4 antibody. Exosomes were more than 150 times stronger in ILNs than in any other tissues, i.e., which means exosomes could effectively target TDLNs after subcutaneous administration and induce more effective anti-tumor T cell responses (Fig. 8b).

Yu’s group [185] compared the accumulation of LNs in four sources of exosomes, RAW264.7 macrophage cell line, mouse serum, human serum, and fetal bovine serum, and tested the ability to act as immune drug carriers to LNs. They found that serum-derived exosomes exhibited better accumulation of LNs than cell-derived exosomes. Fetal bovine serum exosomes could be delivered to the macrophage and T cell areas of the SCS so that immune drugs can target immune cells more precisely. Based on this, their team developed mannose-modified PEGylated bovine serum exosomes (EXO-PEG-man) that were specifically taken up by DCs after intradermal injection and exhibited enhanced lymphatic accumulation, which demonstrates that the exosomes could serve as promising carriers for the delivery of immune drugs or vaccines to LNs (Fig. 8c) [112]. In addition, serum or plasma-derived exosomes have been widely used for the diagnosis of lymphatic metastases from cancers such as pancreatic [186], thyroid [187], and ovarian [188].

Other than that exosomes are from pure origin, there are also research results on artificial chimeric exosomes for targeted drug delivery to LNs inspired by cell fusion into hybrid cells. Wei’s team [114] proposed an LNs and tumor space dual-targeting exosomes. Their team introduced the nucleus of tumor cells into the isolated and activated macrophages in peripheral blood to prepare exosomes. On the one hand, the nano-size of exosomes can be used to passively target LNs and the function inherit from macrophages activates the tumor-specific immune response; on the other hand, the homing ability of tumor cells can be inherited and actively targeted to tumor tissues (Fig. 8d).

Beyond immune cell-derived exosomes, many studies have demonstrated that EVs released from tumor cells can be taken up by lymphatic vessels and transported into draining lymph nodes [189–191]. This phenomenon is attributed to integrins expressed by EVs derived from tumor cells acting as "homing" receptors to direct them to responsive target organs [192]. A study revealed that EVs derived from melanoma cells interacted highly selectively with a subset of macrophages and LECs in TDLNs and that this selectivity was dependent on the expression of lymphoid VCAM-1 (Fig. 8e) [193]. The discovery of this mechanism provides some theoretical basis for the development of tumor-derived EVs as drug carriers targeting LNs.

In conclusion, exosomes or extracellular vesicles of various cellular-derived have their own advantages in targeting LNs and have shown excellent targeting accumulation effects in mouse models. However, exosome isolation is inefficient and industrial production is difficult, so the realization of clinical application is still dependent on the advancement of production technology.

### Microneedles

**Fig. 9:**
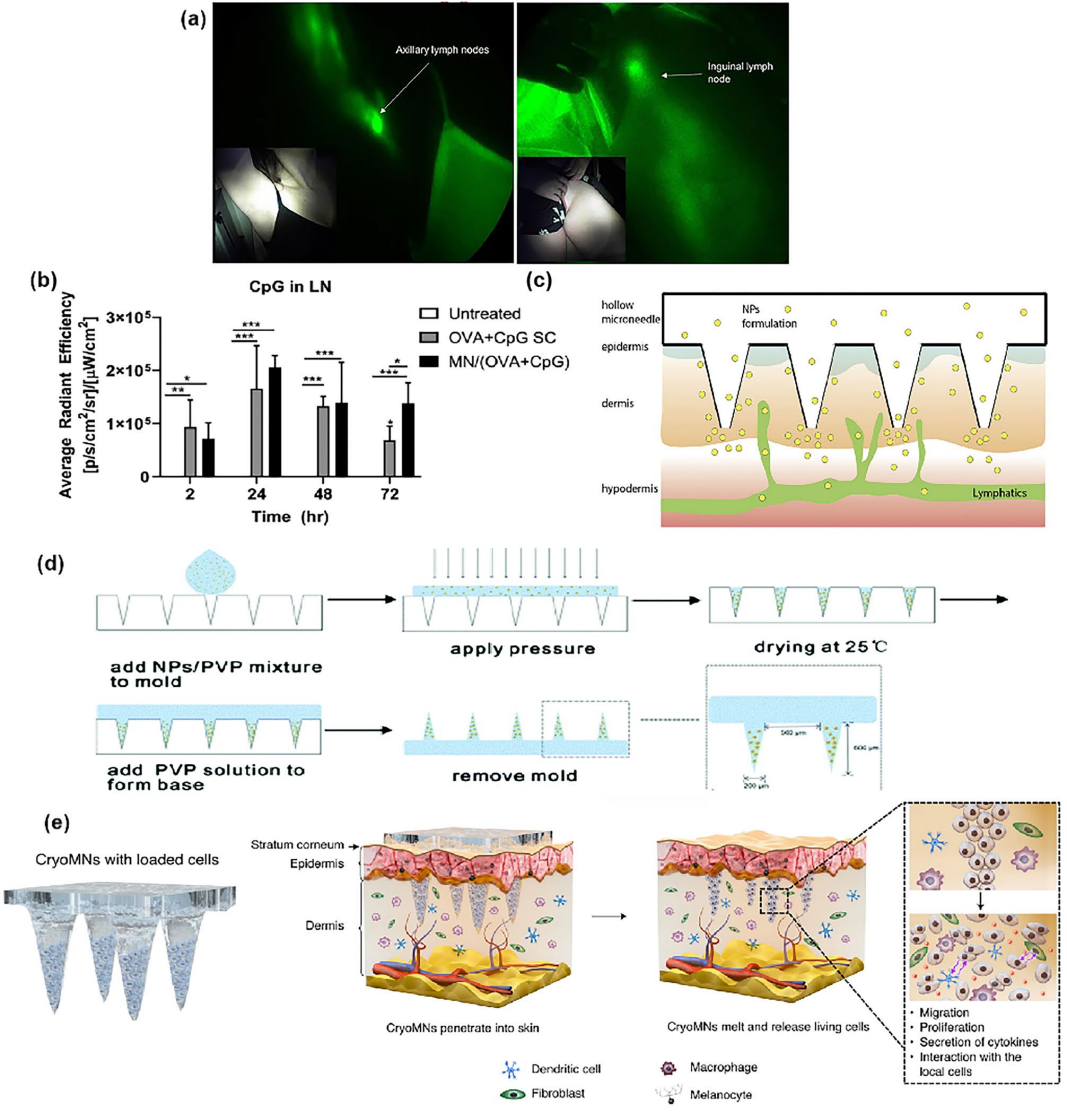
**a** lymphatic vessels pump ICG-laden lymph to the regional ALNs and ILNs [199]. Copyright 2019 Ivyspring International Publisher. **b** CpG average radiant efficiency in draining lymph nodes after treated. Microneedle delivery significantly prolongs the accumulation of OVA and CpG at the site of administration [116]. Copyright 2021 The Proceedings of the National Academy of Sciences. **c** Schematic diagram of PLGA nanoparticles in hollow microneedles released into LNs [201]. Copyright 2018 Elsevier. **d** Schematic illustration of the CS-OVA-CpG loaded soluble microneedles array fabrication process [202]. Copyright 2020 Royal Society of Chemistry. **e** Schematic illustration of the frozen microneedles (left) and it penetrates the skin epidermis and melts then the loaded cells are released (right) [115]. Copyright 2021 Springer Nature

Microneedles are solid or hollow needle-like structures that can deliver drugs to the epidermis or dermis [194, 195]. Microneelines can penetrate the cuticle in a minimally invasive manner and transport cargo to regional lymph capillary vessels [196, 197]. In addition, there are abundant immune cells in the epidermis [198]. These advantageous conditions facilitate the delivery of the cargo to LNs. Kwon et al. [199] found that nanotopography-based microneedle injected into the epidermal space of mice demonstrated in a visualized form that ICG could be absorbed by the initial lymphatic vessels and subsequently pass through the lymphatics into the interior of LNs. In clinical trials, nanotopography-based microneedle was also found to deliver ICG directly to the axillary lymph nodes (ALNs) and ILNs of healthy human volunteers via the lymphatic vessels (Fig. 9a). These results further demonstrate the great potential of microneedle delivery systems for the delivery to LNs. Therefore, the use of microneedles to deliver drugs to LNs to initiate a stronger immune response is a hot topic of research in recent years.

Microneedles, as a macroscale drug delivery system, have a wide range of applications in nanovaccine. The epidermis and dermis are richly distributed with APCs that require only minute amounts of antigenic stimulation to trigger an immune response [200]. Microneedles have been explored to modulate the recruitment, activation, and lymphatic homing effects of APCs [123]. Transdermal immunization instead of conventional immunization has great advantages in terms of improving immune efficiency. The main microneedles used to mediate vaccination are coated microneedles, hollow microneedles, and dissolvable microneedles (Table 4).

**Table 4:** Summary of type of microneedles for LNs immunotherapy

Type of microneedles	Loading drug	Type of cancer	References
Coated microneedles	Trp2 and CpG	/	[204]
Coated microneedles	Poly(I:C) and pOVA	B16-OVA tumor model	[205]
Hollow microneedles	Cold atmospheric plasma and PD-1 antibody	B16F10 tumor model	[203]
Dissolving microneedles	Paclitaxel and Resiquimod	B16F10 tumor model	[206]
Dissolving microneedles	1-MT and PD-1 antibody	B16F10 tumor model	[207]
Dissolving microneedles	Hyaluronate-SIINFEKL (Cytotoxic T-cell epitope peptide)	B16 tumor model	[208]
Dissolving microneedles	Poly(I:C) and pOVA	B16-OVA tumor model	[209]

Coated microneedles can be prepared by applying drugs on the surface of the needle body. For example, Caudill et al. [116] used 3D printing technology to design faceted microneedles with horizontal grooves that significantly enhanced drug loading compared to square conical microneedles. Besides, microneedle delivery significantly prolonged the accumulation of OVA and CpG at the administration site and enhanced the activation of immune cells within LNs compared to subcutaneous injection (Fig. 9b). Hollow microneedles can be obtained by processing solid needle bodies into hollow needle bodies and encapsulating the drug inside the hollow body. Because hollow microneedles often require pressure generated by syringe thrust, it is possible to develop vaccines with dosing, precision and controlled speed. Niu et al. [201] loaded OVA and R837 co-loaded PLGA nanoparticles into hollow microneedle bodies. After administration, nanoparticles are transported through dermal lymphatic vessels and draining lymph nodes in the skin to secondary lymphatic organs (Fig. 9c). Compared to PLGA nanoparticles delivered by intramuscular injection, microneedles were able to significantly enhance Th1 responses, and elicited significantly higher IgG2a antibody responses and more IFN-γ-secreting lymphocytes.

Soluble microneedles are prepared by dissolving or dispersing the drug in easily degradable needle materials, which can slowly degrade in vivo and continuously release the drug. Li et al. [202] used polypyrrolidone (PVP) as a material to load nanoparticles of chitosan-encapsulated OVA and CpG onto needle tips (Fig. 9d). These goods carried by nanoparticles were able to effectively accumulate in peripheral LNs and demonstrate a potent anti-tumor immune response.

Proper microneedle design can also be a carrier for the delivery of live cells. Chang et al. [115] reported a phosphate-buffered solution of 2.5% dimethyl sulfoxide and 100 mM sucrose as a cryogenic medium for loading suspended DCs to construct frozen microneedles. The survival rate of DCs was as high as 71.4 ± 1.4%, and DCs can retain unchanged viability after one month of storage in liquid nitrogen. This provides a novel means of efficient delivery DCs to LNs (Fig. 9e). In addition, due to the specificity of transdermal delivery especially for somatic tumors such as melanoma, microneedles can be used as an adjunct to other treatment modalities. One study described a cold atmospheric plasma (CAP)-mediated ICB therapy integrated with microneedles. Microneedles contributed to CAP transport through the skin leading to tumor antigen release and promoting the maturation of DCs in TDLNs, while the ICB drugs contained in microneedles exerted a synergistic anti-tumor effect [203].

At present, more and more attention has been paid to the research of microneedles, and the emergence of industrialized equipment has also promoted the development of microneedles for drug delivery, like 3D printers. However, there are still some technical obstacles, such as low drug loading, to be broken through in practice.

### Protein Nanoparticles

**Fig. 10:**
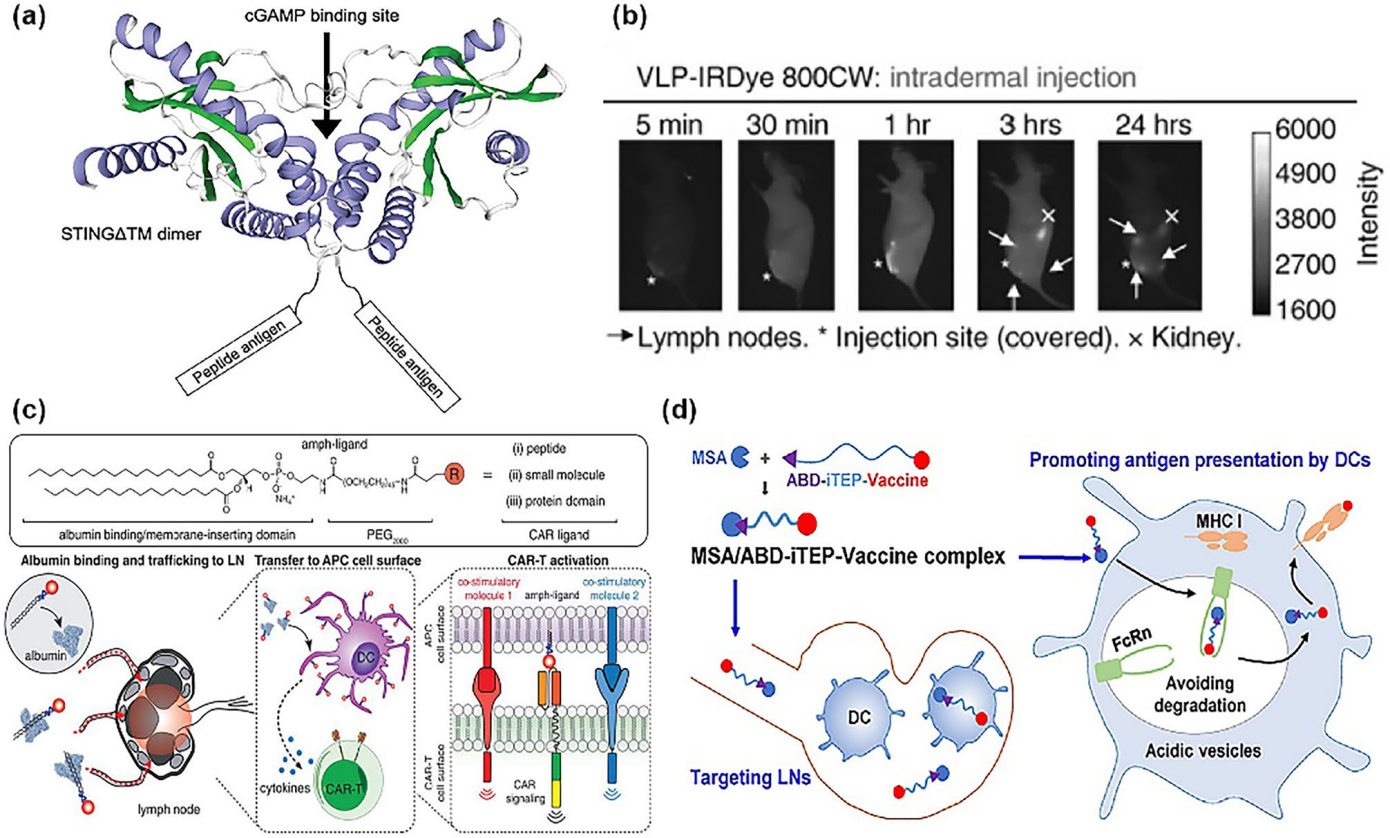
**a** Schematic illustration of the predicted structure of SIINFEKL-STINGΔTM by protein homology modeling [210]. Copyright 2022 John Wiley and Sons. **b** Imaging of SHIV VLP trafficking after immunization by intradermal injection. Fluorescence was detected in multiple LNs, and the specific distribution of fluorescence became more apparent with time [213]. Copyright 2009 Wolters Kluwer Health, Inc. **c** Schematic of the chemical structure of amph-ligands (top) and the steps in amph-ligand vaccine boosting in vivo (bottom) [214]. Copyright 2019 American Association for the Advancement of Science. **d** Schematic illustration of an albumin-binding polypeptide targets LNs and boosts vaccine presentation by DCs [197]. Copyright 2018 Ivyspring International Publisher

Protein nanocarriers have many excellent properties suitable for targeted drug delivery, such as highly organized structure and symmetry, good biocompatibility and biodegradability, and ideal delivery size [117]. Based on current developments in proteomics, recombinant protein engineering, and other technologies that confer predictability, tunability, and designability to protein carriers. He [210] complexed and self-assembled a peptide, interferon gene stimulator (STINGΔTM) protein, and STING agonist 2′3′cyclic guanosine monophosphate-adenosine monophosphate (cGAMP) into a tetramer (Fig. 10a). cGAMP-STINGΔTM served as a protein carrier for the peptide and activated STING signaling to aid in the activation of APCs. Experiment results showed that the tetramer exhibited excellent lymphatic targeting efficacy.

Another protein-based carrier of interest is virus-like particles (VLPs), which are hollow viral capsids formed by single or multiple structural proteins but lacking a nucleic acid component. It allows the highly repetitive display of natural antigens on the surface of VLPs used to activate immune cells nor to infect the organism. Coupled with the favorable size of VLPs (20–200 nm) allows them to be freely diverted in their intact form to LNs [211, 212]. It was shown back in 2009 that 90 nm VLPs could easily enter the subcapsular sinus of draining lymph nodes. Their trials have shown that the simian-human immunodeficiency virus (SHIVs) showed strong accumulation in multiple LNs, particularly when given with an intradermal injection. This phenomenon increased titers of higher affinity antibodies (Fig. 10b) [213].

In addition to the use of exogenous proteins for the delivery of LNs, the "albumin hitchhiking" strategy can enhance the efficiency of LNs delivery by using albumin. Albumin exceeds the threshold for diffusion from the interstitial space into the bloodstream and is trafficked to the lymphatics to target LNs [64]. The first application of this targeting strategy to a vaccine was that Irvine designed an amphiphilic adjuvant “amph-CpG” linked with a lipophilic albumin-binding region (diacyl lipid), which can bind to endogenous albumin when co-delivered with antigen, thereby enhancing LNs targeting [100]. Same principle, Ma et al. [214] used this approach to create enhanced vaccines for CAR-T by designing "amph-ligands" that attach CAR ligands to polymer-lipid tails which can ride on albumin, exhibiting LNs targeting CAR-T cells rapid activation and efficient proliferation (Fig. 10c).

In addition to the natural transport of cargo to LNs achieved with the help of lipid-soluble albumin-binding structural domains, using the specific binding of the albumin-binding structural domain and serum albumin also can do it. Wang et al. [215] designed a recombinant fusion protein consisting of an albumin-binding domain (ABD), an immune-tolerant elastin-like polypeptide (iTEP), and an antigenic peptide. The association between ABD-iTEP and mouse serum albumin (MSA) in mice exhibited better accumulation of LNs and accumulation of DCs, facilitating antigen presentation provided by ABD-iTEP (Fig. 10d). Of course, this albumin-binding domain can also be modified on other carriers to confer this particular transport strategy. A study described the modification of ABD on the surface of extracellular vesicles. It was demonstrated that the modification exerted a strong binding capacity with human serum albumin (HSA) and MSA. This design significantly increased the cycle time of EVs and showed a considerable accumulation of LNs and solid tumors [216].

In conclusion, the application of both exogenous protein carriers and endogenous albumin is focused on tumor vaccines. Because they have an outstanding advantage in boosting the immune response. Protein carriers are expected to participate in the combination therapy of cancer in clinical treatment [117].

### Others

In addition to the above classical carriers, thanks to the development and contribution of various cross-disciplinary disciplines, innovative means of achieving targeted drug transport have emerged in recent years, and clever carrier designs have suggested new ideas for LNs accumulation.

**Fig. 11:**
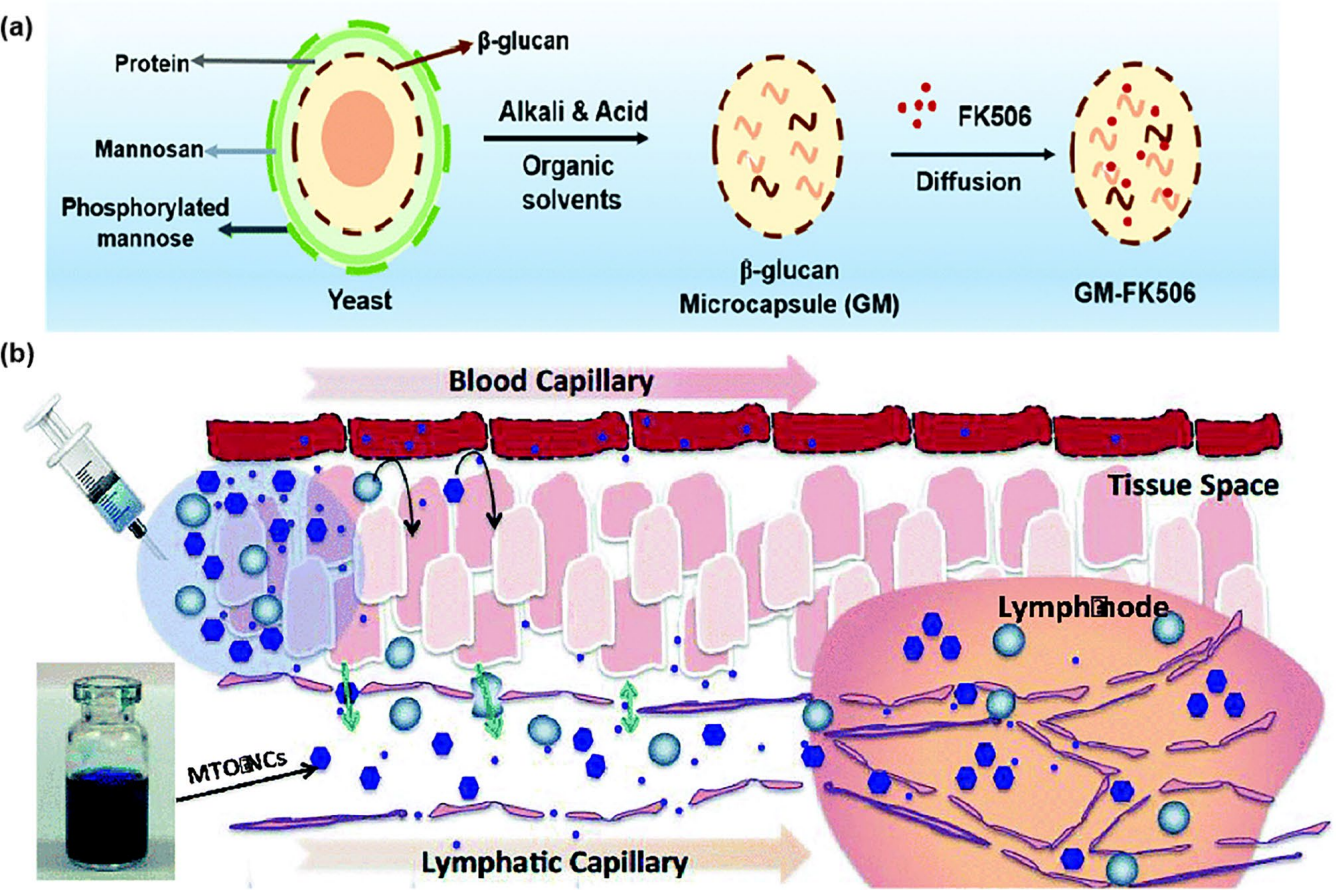
**a** Schematic representation of GM-FK506 preparation [119]. Copyright 2019 Springer Nature. **b** Schematic illustration of MTO NCs to lymphatic targeting process (Gray balls: MTO liposomes, blue dots: MTO molecular and dark blue hexagons: MTO NCs) [118]. Copyright 2020 Royal Society of Chemistry. (Color figure online)

Microbial cancer therapeutics are of great interest, where β-glucan and chitin in yeast cell walls act as "danger signal" leading to immune activation, such as activation of APCs to enhance the anti-tumor efficacy of T cells. Based on this, the team of Wang [74] created cell wall nanoparticles derived from saccharomyces cerevisiae. Wall nanoparticles were effectively recognized by DCs after intratumoral injection by size effect. And were able to efficiently migrate to nearby TDLNs, to activate immune cells and reshape the immunosuppressive tumor microenvironment. Also inspired by the fungal infection pathway, Wu et al. [119] reported an oral β-glucan microcapsules (GM-FK506) (Fig. 11a). The microcapsules are recognized by micro-fold cells with high expression of the Dectin-1 receptor and transported to enteric-related lymphoid tissue. Then microcapsules were phagocytosed by macrophages in this lymphoid tissue, enabling efficient delivery of the contained drug to LNs. Although this study was used to deliver immunosuppressive drugs to treat acute rejection after heart transplantation, it provides meaningful insights into oral LNs targeting systems.

The ideal lymphatic targeting ability of nanocrystals (NCs) formed by their size, and their solution form has better mobility and a faster targeting rate. One study reported that MTO self-assembled nanocrystals (MTO NCs) showed amazing targeting ability for LNs, with 81 times higher uptake of MTO in LNs than in tissues such as the heart, liver, spleen, lung, and kidney (Fig. 11b). Not only that, MTO NCs allowed sustained drug release for up to 24 h and reduced drug toxicity [118].

It is helpful to explore non-classical nanocarriers to prepare LNs targeted delivery systems. This promises to overcome problems encountered with ordinary nanocarriers and achieve greater efficacy.

## Conclusion

Tumor immunotherapy has been hailed as the third revolution in cancer treatment and has transformed the future direction of tumor therapy. LNs coordinate antigen presentation necessary for anti-tumor immunity and become a key target for tumor immunotherapy. Poor immune functional compound delivery in the LNs undoubtedly limits the rapid induction and activation of immune cells, and well-designed drug delivery vectors are an attractive strategy to face this challenge head-on. Current carriers design to achieve effective accumulation of LNs focus on four aspects: first, size effect. According to LNs structure, designing a suitable size aims to overcome the barrier of LNs delivery for passive targeting, which has become the primary design factor to achieve efficient LNs accumulation in different nano-delivery systems. Secondly, active targeting. The active targeting potential of carriers by modifying ligands (e.g., mannose ligands and CD11b antibodies) can significantly improve the accumulation of LNs in nano-delivery systems. It can be widely used in the design of different kinds of nano-delivery systems. Third, functionalized carriers. It can be achieved in this way that relying on the special physiological environment, synthetic chemical bonds, or special materials to make carriers undergo the predicted chemical reactions in vivo by using the carrier's own chemical properties. The construction of functionalized carriers with modifications such as pH sensitivity, oxidation triggering, the introduction of the linker, and click chemistry, allows for modulation of particle size, precise drug release, or enhanced target delivery. Fourth, the use of advanced biotechnology. On the basis of retaining the advantages of the original delivery carriers, carriers are cleverly transformed with the help of bionic technology, protein fusion technology, recombinant protein engineering, genetic engineering, etc. The incorporation of new technologies continues to suggest interesting ideas for the design of LNs targeting carriers.

Although different types of nanocarriers present different advantages for the targeted delivery of LNs, and delivery systems with new optimized mechanisms and high delivery efficacy are being developed, these approaches still face the challenge of clinical translation. Many nanomedicine products show good results in cell and animal experiments, however, the results of clinical research are not satisfactory, especially for the delivery system with site-specific accumulation. Firstly, the efficacy of nano-delivery carriers in humans has not been fully validated. The pharmacokinetics in vivo are not easily elucidated. After injection, the nanoparticles do not precisely target to LNs in the designed path. It is inevitable to accumulate in the organs such as the liver and spleen, then leading to toxicity to normal tissues. Besides, ensuring the safety of the materials is a great obstacle for clinical translation. Although there are many types of carriers used for the targeted delivery of LNs, only a few types are used in clinical applications. How to enhance the biocompatibility and biodegradability of carriers in vivo is one of the main issues for clinical translation. Thirdly, the technical challenges of production are critical for clinical translation. The sample preparation technology is a prerequisite for the clinical translation of the nano-delivery systems.

In conclusion, selective delivery of drugs to LNs may allow us to overcome the limitations of existing tumor immunotherapies and is therefore essential for the advancement of immunotherapy. However, the development of nanoparticles for LNs with high safety, simple preparation process, easy industrial production and potential for clinical translation is not only limited to the development of pharmaceutics, but also depends on the integration of interdisciplinary technologies such as medicine, biology, chemistry and mechanical science. Industrial pharmaceutics, materials science, mechanical engineering, molecular biology, protein engineering and so on are the key point to guarantee the clinical transformation of LNs tumor immunotherapy.

## References

[1] Chouaib S, El Hage F, Benlalam H, Mami-Chouaib F (2006). Immunotherapy of cancer: promise and reality. MS Méd. Sci..

[2] Shi Y, Lammers T (2019). Combining nanomedicine and immunotherapy. Acc. Chem. Res..

[3] Caldwell KJ, Gottschalk S, Talleur AC (2021). Allogeneic car cell therapy—more than a pipe dream. Front. Immunol..

[4] Lesterhuis WJ, Haanen JBAG, Punt CJA (2011). Cancer immunotherapy-revisited. Nat. Rev. Drug Disc..

[5] Xie X, Song T, Feng Y, Zhang H, Yang G (2022). Nanotechnology-based multifunctional vaccines for cancer immunotherapy. Chem. Eng. J..

[6] Burugu S, Dancsok AR, Nielsen TO (2018). Emerging targets in cancer immunotherapy. Semin. Cancer Biol..

[7] Jørgensen J, Hanna E, Kefalas P (2020). Outcomes-based reimbursement for gene therapies in practice: the experience of recently launched car-t cell therapies in major european countries. J. Market Access Health Policy.

[8] Morad G, Helmink BA, Sharma P, Wargo JA (2021). Hallmarks of response, resistance, and toxicity to immune checkpoint blockade. Cell.

[9] Hegde PS, Chen DS (2020). Top 10 challenges in cancer immunotherapy. Immunity.

[10] Boehm T, Bleul CC (2007). The evolutionary history of lymphoid organs. Nat. Immunol..

[11] S. Sell, How the immune system works. Medical Times **108**(12), 60–63, 67–68, 70–61 passim (1980) 6969341

[12] von Andrian UH, Mempel TR (2003). Homing and cellular traffic in lymph nodes. Nat. Rev. Immunol..

[13] Wang L, Subasic C, Minchin RF, Kaminskas LM (2019). Drug formulation and nanomedicine approaches to targeting lymphatic cancer metastases. Nanomedicine.

[14] Albalawi F, Hussein MZ, Fakurazi S, Masarudin MJ (2021). Engineered nanomaterials: the challenges and opportunities for nanomedicines. Int. J. Nanomed..

[15] Dadwal A, Baldi A, Kumar Narang R (2018). Nanoparticles as carriers for drug delivery in cancer. Artif. Cells Nanomed. Biotechnol..

[16] Flores de los Rios PA, Casañas Pimentel RG, San Martín Martínez E (2022). Nanodrugs against cancer: biological considerations in its redesign. Int. J. Polym. Mater..

[17] Dong P, Rakesh KP, Manukumar HM, Mohammed YHE, Karthik CS (2019). Innovative nano-carriers in anticancer drug delivery-a comprehensive review. Bioorg. Chem..

[18] Schudel A, Francis DM, Thomas SN (2019). Material design for lymph node drug delivery. Nat. Rev. Mater..

[19] Trac N, Chung EJ (2021). Overcoming physiological barriers by nanoparticles for intravenous drug delivery to the lymph nodes. Exper. Biol. Med..

[20] Najibi AJ, Mooney DJ (2020). Cell and tissue engineering in lymph nodes for cancer immunotherapy. Adv. Drug Deliv. Rev..

[21] Berg EL, Robinson MK, Warnock RA, Butcher EC (1991). The human peripheral lymph node vascular addressin is a ligand for lecam-1, the peripheral lymph node homing receptor. J. Cell Biol..

[22] Ruddle NH, Akirav EM (2009). Secondary lymphoid organs: responding to genetic and environmental cues in ontogeny and the immune response1. J. Immun..

[23] Chen Y, De Koker S, De Geest BG (2020). Engineering strategies for lymph node targeted immune activation. Acc. Chem. Res..

[24] Ding Y, Li Z, Jaklenec A, Hu Q (2021). Vaccine delivery systems toward lymph nodes. Adv. Drug Deliv. Rev..

[25] Bajénoff M, Egen JG, Koo LY, Laugier JP, Brau F (2006). Stromal cell networks regulate lymphocyte entry, migration, and territoriality in lymph nodes. Immunity.

[26] Manspeaker MP, Thomas SN (2020). Lymphatic immunomodulation using engineered drug delivery systems for cancer immunotherapy. Adv. Drug Deliv. Rev..

[27] Girard J-P, Moussion C, Förster R (2012). Hevs, lymphatics and homeostatic immune cell trafficking in lymph nodes. Nat. Rev. Immunol..

[28] Zhang Y-N, Poon W, Sefton E, Chan WCW (2020). Suppressing subcapsular sinus macrophages enhances transport of nanovaccines to lymph node follicles for robust humoral immunity. ACS Nano.

[29] Swartz MA (2001). The physiology of the lymphatic system. Adv. Drug Deliv. Rev..

[30] du Bois H, Heim TA, Lund AW (2021). Tumor-draining lymph nodes: at the crossroads of metastasis and immunity. Sci. Immunol..

[31] Das S, Sarrou E, Podgrabinska S, Cassella M, Mungamuri SK (2013). Tumor cell entry into the lymph node is controlled by ccl1 chemokine expressed by lymph node lymphatic sinuses. J. Exp. Med..

[32] Gasteiger G, Ataide M, Kastenmüller W (2016). Lymph node-an organ for t-cell activation and pathogen defense. Immunol. Rev..

[33] Pereira ER, Jones D, Jung K, Padera TP (2015). The lymph node microenvironment and its role in the progression of metastatic cancer. Sem. Cell Dev. Biol..

[34] Lian J, Luster AD (2015). Chemokine-guided cell positioning in the lymph node orchestrates the generation of adaptive immune responses. Curr. Opin. Cell Biol..

[35] Wendland M, Willenzon S, Kocks J, Davalos-Misslitz AC, Hammerschmidt SI (2011). Lymph node t cell homeostasis relies on steady state homing of dendritic cells. Immunity.

[36] Katakai T, Hara T, Lee J-H, Gonda H, Sugai M (2004). A novel reticular stromal structure in lymph node cortex: an immuno-platform for interactions among dendritic cells, t cells and b cells. Int. Immunol..

[37] Willard-Mack CL (2006). Normal structure, function, and histology of lymph nodes. Tox. Pathol..

[38] Ohtani O, Ohtani Y (2008). Structure and function of rat lymph nodes. Arch. Histol. Cytol..

[39] O'Neill NA, Eppler HB, Jewell CM, Bromberg JS (2018). Harnessing the lymph node microenvironment. Curr. Opin. Organ Transpl..

[40] Radomski M, Zeh HJ, Edington HD, Pingpank JF, Butterfield LH (2016). Prolonged intralymphatic delivery of dendritic cells through implantable lymphatic ports in patients with advanced cancer. J. Immunother. Cancer.

[41] Fujii H, Horie S, Takeda K, Mori S, Kodama T (2018). Optimal range of injection rates for a lymphatic drug delivery system. J. Biophotonics.

[42] Jiang H, Wang Q, Sun X (2017). Lymph node targeting strategies to improve vaccination efficacy. J. Contr. Release.

[43] von Beust BR, Johansen P, Smith KA, Bot A, Storni T (2005). Improving the therapeutic index of cpg oligodeoxynucleotides by intralymphatic administration. Eur. J. Immunol..

[44] Lesterhuis WJ, De Vries IJM, Schreibelt G, Lambeck AJA, Aarntzen EHJG (2011). Route of administration modulates the induction of dendritic cell vaccine-induced antigen-specific t cells in advanced melanoma patients. Clin. Cancer Res..

[45] Johansen P, Häffner AC, Koch F, Zepter K, Erdmann I (2005). Direct intralymphatic injection of peptide vaccines enhances immunogenicity. Eur. J. Immunol..

[46] Jewell CM, Bustamante López SC, Irvine DJ (2011). In situ engineering of the lymph node microenvironment via intranodal injection of adjuvant-releasing polymer particles. Proc. Natl. Acad. Sci. USA.

[47] Choi CH, Zuckerman JE, Webster P, Davis ME (2011). Targeting kidney mesangium by nanoparticles of defined size. Proc. Natl. Acad. Sci. USA.

[48] Alexis F, Pridgen E, Molnar LK, Farokhzad OC (2008). Factors affecting the clearance and biodistribution of polymeric nanoparticles. Mol. Pharm..

[49] Rodrigues SF, Granger DN (2015). Blood cells and endothelial barrier function. Tissue Barriers.

[50] Engin AB, Nikitovic D, Neagu M, Henrich-Noack P, Docea AO (2017). Mechanistic understanding of nanoparticles' interactions with extracellular matrix: the cell and immune system. Particle Fibre Toxicol..

[51] Kaminskas LM, McLeod VM, Ascher DB, Ryan GM, Jones S (2015). Methotrexate-conjugated pegylated dendrimers show differential patterns of deposition and activity in tumor-burdened lymph nodes after intravenous and subcutaneous administration in rats. Mol. Pharm..

[52] Chida T, Miura Y, Cabral H, Nomoto T, Kataoka K (2018). Epirubicin-loaded polymeric micelles effectively treat axillary lymph nodes metastasis of breast cancer through selective accumulation and ph-triggered drug release. J. Contr. Release.

[53] Xia C, Zhou Q, Zhang Q, Hu S, Meacci E (2021). Comparative study on the diagnostic value of intravenous/peritumoral injection of indocyanine green for metastatic lymph node location in patients with head and neck squamous cell carcinoma (hnscc). Ann. Transl. Med..

[54] Trevaskis NL, Kaminskas LM, Porter CJH (2015). From sewer to saviour-targeting the lymphatic system to promote drug exposure and activity. Nat. Rev. Drug Disc..

[55] Trevaskis NL, Charman WN, Porter CJH (2010). Targeted drug delivery to lymphocytes: a route to site-specific immunomodulation?. Mol. Pharm..

[56] Vela Ramirez JE, Sharpe LA, Peppas NA (2017). Current state and challenges in developing oral vaccines. Adv. Drug Deliv. Rev..

[57] Azizi A, Kumar A, Diaz-Mitoma F, Mestecky J (2010). Enhancing oral vaccine potency by targeting intestinal m cells. PLoS Pathog..

[58] Florence AT (2005). Nanoparticle uptake by the oral route: fulfilling its potential?. Drug Disc. Today Technol..

[59] Yáñez JA, Wang SW, Knemeyer IW, Wirth MA, Alton KB (2011). Intestinal lymphatic transport for drug delivery. Adv. Drug Deliv. Rev..

[60] Hu Q, Wu M, Fang C, Cheng C, Zhao M (2015). Engineering nanoparticle-coated bacteria as oral DNA vaccines for cancer immunotherapy. Nano Lett..

[61] Ryan GM, Kaminskas LM, Porter CJH (2014). Nano-chemotherapeutics: maximising lymphatic drug exposure to improve the treatment of lymph-metastatic cancers. J. Contr. Release.

[62] Feng L, Zhang L, Liu M, Yan Z, Wang C (2010). Roles of dextrans on improving lymphatic drainage for liposomal drug delivery system. J. Drug Target..

[63] Nicolas JF, Guy B (2008). Intradermal, epidermal and transcutaneous vaccination: from immunology to clinical practice. Expert Rev. Vaccines.

[64] Hoogenboezem EN, Duvall CL (2018). Harnessing albumin as a carrier for cancer therapies. Adv. Drug Deliv. Rev..

[65] Rohner NA, Thomas SN (2017). Flexible macromolecule versus rigid particle retention in the injected skin and accumulation in draining lymph nodes are differentially influenced by hydrodynamic size. ACS BioMater. Sci. Eng..

[66] Wang Y, Wang J, Zhu D, Wang Y, Qing G (2021). Effect of physicoChemical properties on in vivo fate of nanoparticle-based cancer immunotherapies. Acta Pharm. Sin. B.

[67] Chaturvedi S, Garg A, Verma A (2020). Nano lipid based carriers for lymphatic voyage of anti-cancer drugs: an insight into the in-vitro, ex-vivo, in-situ and in-vivo study models. J. Drug Deliv. Sci. Technol..

[68] Nakamura T, Kawai M, Sato Y, Maeki M, Tokeshi M (2020). The effect of size and charge of lipid nanoparticles prepared by microfluidic mixing on their lymph node transitivity and distribution. Mol. Pharm..

[69] Reddy ST, van der Vlies AJ, Simeoni E, Angeli V, Randolph GJ (2007). Exploiting lymphatic transport and complement activation in nanoparticle vaccines. Nat. Biotechnol..

[70] He R, Zang J, Zhao Y, Dong H, Li Y (2022). Nanotechnology-based approaches to promote lymph node targeted delivery of cancer vaccines. ACS Biomater. Sci. Eng..

[71] Yu X, Dai Y, Zhao Y, Qi S, Liu L (2020). Melittin-lipid nanoparticles target to lymph nodes and elicit a systemic anti-tumor immune response. Nat. Commun..

[72] Bachmann MF, Jennings GT (2010). Vaccine delivery: a matter of size, geometry, kinetics and molecular patterns. Nat. Rev. Immunol..

[73] Manolova V, Flace A, Bauer M, Schwarz K, Saudan P (2008). Nanoparticles target distinct dendritic cell populations according to their size. Eur. J. Immunol..

[74] Xu J, Ma Q, Zhang Y, Fei Z, Sun Y (2022). Yeast-derived nanoparticles remodel the immunosuppressive microenvironment in tumor and tumor-draining lymph nodes to suppress tumor growth. Nat. Commun..

[75] Albanese A, Tang PS, Chan WCW (2012). The effect of nanoparticle size, shape, and surface chemistry on biological systems. Ann. Rev. Biomed. Eng..

[76] Zeng Q, Jiang H, Wang T, Zhang Z, Gong T (2015). Cationic micelle delivery of trp2 peptide for efficient lymphatic draining and enhanced cytotoxic t-lymphocyte responses. J. Contr. Release.

[77] Zhuang Y, Ma Y, Wang C, Hai L, Yan C (2012). Pegylated cationic liposomes robustly augment vaccine-induced immune responses: role of lymphatic trafficking and biodistribution. J. Contr. Release.

[78] Nakamura T, Harashima H (2020). Dawn of lipid nanoparticles in lymph node targeting: potential in cancer immunotherapy. Adv. Drug Deliv. Rev..

[79] McCright J, Skeen C, Yarmovsky J, Maisel K (2022). Nanoparticles with dense poly(ethylene glycol) coatings with near neutral charge are maximally transported across lymphatics and to the lymph nodes. Acta Biomater..

[80] Zou Y, Ito S, Yoshino F, Suzuki Y, Zhao L (2020). Polyglycerol grafting shields nanoparticles from protein corona formation to avoid macrophage uptake. ACS Nano.

[81] Zhan X, Tran KK, Shen H (2012). Effect of the poly(ethylene glycol) (peg) density on the access and uptake of particles by antigen-presenting cells (apcs) after subcutaneous administration. Mol. Pharm..

[82] Alvarez D, Vollmann EH, von Andrian UH (2008). Mechanisms and consequences of dendritic cell migration. Immunity.

[83] Song T, Xia Y, Du Y, Chen MW, Qing H (2021). Engineering the deformability of albumin-stabilized emulsions for lymph-node vaccine delivery. Adv. Mater..

[84] Guo P, Liu D, Subramanyam K, Wang B, Yang J (2018). Nanoparticle elasticity directs tumor uptake. Nat. Commun..

[85] Key J, Palange AL, Gentile F, Aryal S, Stigliano C (2015). Soft discoidal polymeric nanoconstructs resist macrophage uptake and enhance vascular targeting in tumors. ACS Nano.

[86] Mueller SN, Tian S, DeSimone JM (2015). Rapid and persistent delivery of antigen by lymph node targeting print nanoparticle vaccine carrier to promote humoral immunity. Mol. Pharm..

[87] Cai T, Liu H, Zhang S, Hu J, Zhang L (2021). Delivery of nanovaccine towards lymphoid organs: recent strategies in enhancing cancer immunotherapy. J. Nanobiotechn..

[88] Macri C, Dumont C, Johnston AP, Mintern JD (2016). Targeting dendritic cells: a promising strategy to improve vaccine effectiveness. Clin. Transl. Immunol..

[89] Keler T, Ramakrishna V, Fanger MW (2004). Mannose receptor-targeted vaccines. Expert Opin. Biol. Ther..

[90] Duinkerken S, Horrevorts SK, Kalay H, Ambrosini M, Rutte L (2019). Glyco-dendrimers as intradermal anti-tumor vaccine targeting multiple skin DC subsets. Theranostics.

[91] Duluc D, Joo H, Ni L, Yin W, Upchurch K (2014). Induction and activation of human th17 by targeting antigens to dendritic cells via dectin-1. J. Immunol..

[92] Gehrie E, Van der Touw W, Bromberg JS, Ochando JC (2011). Plasmacytoid dendritic cells in tolerance. Methods Mol. Biol..

[93] Le Moignic A, Malard V, Benvegnu T, Lemiègre L, Berchel M (2018). Preclinical evaluation of mrna trimannosylated lipopolyplexes as therapeutic cancer vaccines targeting dendritic cells. J. Contr. Release.

[94] Li X, Khorsandi S, Wang Y, Santelli J, Huntoon K (2022). Cancer immunotherapy based on image-guided sting activation by nucleotide nanocomplex-decorated ultrasound microbubbles. Nat. Nanotechnol..

[95] Rohner NA, McClain J, Tuell SL, Warner A, Smith B (2015). Lymph node biophysical remodeling is associated with melanoma lymphatic drainage. FASEB J..

[96] Balachandran YL, Li X, Jiang X (2021). Integrated microfluidic synthesis of aptamer functionalized biozeolitic imidazolate framework (bioZIF-8) targeting lymph node and tumor. Nano Lett..

[97] Liu H, Wen Z, Chen H, Yang Z, Le Z (2022). Nanoadjuvants actively targeting lymph node conduits and blocking tumor invasion in lymphatic vessels. J. Contr. Release.

[98] Jiang L, Jung S, Zhao J, Kasinath V, Ichimura T (2021). Simultaneous targeting of primary tumor, draining lymph node, and distant metastases through high endothelial venule-targeted delivery. Nano Today.

[99] Qin H, Zhao R, Qin Y, Zhu J, Chen L (2021). Development of a cancer vaccine using in vivo click-chemistry-mediated active lymph node accumulation for improved immunotherapy. Adv. Mater..

[100] Liu H, Moynihan KD, Zheng Y, Szeto GL, Li AV (2014). Structure-based programming of lymph-node targeting in molecular vaccines. Nature.

[101] Stephan MT, Moon JJ, Um SH, Bershteyn A, Irvine DJ (2010). Therapeutic cell engineering with surface-conjugated synthetic nanoparticles. Nat. Med..

[102] Yang P, Song H, Qin Y, Huang P, Zhang C (2018). Engineering dendritic-cell-based vaccines and pd-1 blockade in self-assembled peptide nanofibrous hydrogel to amplify antitumor t-cell immunity. Nano Lett..

[103] Meng Z, Zhang Y, She J, Zhou X, Xu J (2021). Ultrasound-mediated remotely controlled nanovaccine delivery for tumor vaccination and individualized cancer immunotherapy. Nano Lett..

[104] Roces CB, Khadke S, Christensen D, Perrie Y (2019). Scale-independent microfluidic production of cationic liposomal adjuvants and development of enhanced lymphatic targeting strategies. Mol. Pharm..

[105] Du Y, Song T, Wu J, Gao X-D, Ma G (2022). Engineering mannosylated pickering emulsions for the targeted delivery of multicomponent vaccines. Biomaterials.

[106] Mei L, Rao J, Liu Y, Li M, Zhang Z (2018). Effective treatment of the primary tumor and lymph node metastasis by polymeric micelles with variable particle sizes. J. Contr. Release.

[107] Xiao P, Wang J, Zhao Z, Liu X, Sun X (2021). Engineering nanoscale artificial antigen-presenting cells by metabolic dendritic cell labeling to potentiate cancer immunotherapy. Nano Lett..

[108] Wang Q, Dong Z, Lou F, Yin Y, Zhang J (2022). Phenylboronic ester-modified polymeric nanoparticles for promoting trp2 peptide antigen delivery in cancer immunotherapy. Drug Deliv..

[109] Huang C, Zhang L, Guo Q, Zuo Y, Wang N (2021). Robust nanovaccine based on polydopamine-coated mesoporous silica nanoparticles for effective photothermal-immunotherapy against melanoma. Adv. Funct. Mater..

[110] Zhong X, Zhang Y, Tan L, Zheng T, Hou Y, Hong X, Du G, Chen X, Zhang Y, Sun X (2019). An aluminum adjuvant-integrated nano-mof as antigen delivery system to induce strong humoral and cellular immune responses. J. Contr. Release.

[111] Sun I-C, Jo S, Dumani D, Yun WS, Yoon HY (2021). Theragnostic glycol chitosan-conjugated gold nanoparticles for photoacoustic imaging of regional lymph nodes and delivering tumor antigen to lymph nodes. Nanomaterials.

[112] Choi ES, Song J, Kang YY, Mok H (2019). Mannose-modified serum exosomes for the elevated uptake to murine dendritic cells and lymphatic accumulation. Macromol. Biosci..

[113] Zuo B, Zhang Y, Zhao K, Wu L, Qi H (2022). Universal immunotherapeutic strategy for hepatocellular carcinoma with exosome vaccines that engage adaptive and innate immune responses. J. Hematol. Oncol..

[114] Wang S, Li F, Ye T, Wang J, Lyu C (2021). Macrophage-tumor chimeric exosomes accumulate in lymph node and tumor to activate the immune response and the tumor microenvironment. Sci. Transl. Med..

[115] Chang H, Chew SWT, Zheng M, Lio DCS, Wiraja C (2021). Cryomicroneedles for transdermal cell delivery. Nat. Biomed. Eng..

[116] Caudill C, Perry JL, Iliadis K, Tessema AT, Lee BJ (2021). Transdermal vaccination via 3d-printed microneedles induces potent humoral and cellular immunity. Proc. Natl. Acad. Sci..

[117] Neek M, Kim TI, Wang S-W (2019). Protein-based nanoparticles in cancer vaccine development. Nanomed. Nanotechnol. Biol. Med..

[118] Mao Y, Liu J, Shi T, Chen G, Wang S (2019). A novel self-assembly nanocrystal as lymph node-targeting delivery system: higher activity of lymph node targeting and longer efficacy against lymphatic metastasis. AAPS Pharm. Sci. Tech..

[119] Wu Y, Jin Q, Chen Y, Li H, Deng C (2020). Bioinspired ß-glucan microcapsules deliver FK506 to lymph nodes for treatment of cardiac allograft acute rejection. BioMater. Sci..

[120] Kashyap N, Kumar N, Kumar MNVR (2005). Hydrogels for pharmaceutical and biomedical applications. Cri. Rev. Ther. Drug Carrier Syst..

[121] Chao Y, Chen Q, Liu Z (2020). Smart injectable hydrogels for cancer immunotherapy. Adv. Funct. Mater..

[122] Cirillo G, Spizzirri UG, Curcio M, Nicoletta FP, Iemma F (2019). Injectable hydrogels for cancer therapy over the last decade. Pharmaceutics.

[123] Wang J, Wang S, Ye T, Li F, Gao X (2020). Choice of nanovaccine delivery mode has profound impacts on the intralymph node spatiotemporal distribution and immunotherapy efficacy. Adv. Sci..

[124] Song H, Huang P, Niu J, Shi G, Zhang C (2018). Injectable polypeptide hydrogel for dual-delivery of antigen and TLR3 agonist to modulate dendritic cells in vivo and enhance potent cytotoxic t-lymphocyte response against melanoma. Biomaterials.

[125] Ding M, Fan Y, Lv Y, Liu J, Yu N (2022). A prodrug hydrogel with tumor microenvironment and near-infrared light dual-responsive action for synergistic cancer immunotherapy. Acta Biomater..

[126] Wang H, Najibi AJ, Sobral MC, Seo BR, Lee JY (2020). Biomaterial-based scaffold for in situ chemo-immunotherapy to treat poorly immunogenic tumors. Nat. Commun..

[127] Pérez del Río E, Santos F, Rodriguez Rodriguez X, Martínez-Miguel M, Roca-Pinilla R (2020). CCL21-loaded 3D hydrogels for t cell expansion and differentiation. Biomaterials.

[128] Shao Y, Sun Z-Y, Wang Y, Zhang B-D, Liu D (2018). Designable immune therapeutical vaccine system based on DNA supramolecular hydrogels. ACS Appl. Mater. Interfaces.

[129] Sun L, Shen F, Tian L, Tao H, Xiong Z (2021). Atp-responsive smart hydrogel releasing immune adjuvant synchronized with repeated chemotherapy or radiotherapy to boost antitumor immunity. Adv. Mater..

[130] Nguyen TL, Cha BG, Choi Y, Im J, Kim J (2020). Injectable dual-scale mesoporous silica cancer vaccine enabling efficient delivery of antigen/adjuvant-loaded nanoparticles to dendritic cells recruited in local macroporous scaffold. Biomaterials.

[131] Su Q, Song H, Huang P, Zhang C, Yang J (2021). Supramolecular co-assembly of self-adjuvanting nanofibrious peptide hydrogel enhances cancer vaccination by activating myd88-dependent nf-κb signaling pathway without inflammation. Bioactive Mater..

[132] Kamalov M, Kählig H, Rentenberger C, Müllner ARM, Peterlik H (2019). Ovalbumin epitope siinfekl self-assembles into a supramolecular hydrogel. Sci. Rep..

[133] Song H, Yang P, Huang P, Zhang C, Kong D (2019). Injectable polypeptide hydrogel-based co-delivery of vaccine and immune checkpoint inhibitors improves tumor immunotherapy. Theranostics.

[134] Wu Y, Li Q, Shim G, Oh Y-K (2021). Melanin-loaded cpg DNA hydrogel for modulation of tumor immune microenvironment. J. Contr. Release.

[135] Umeki Y, Mohri K, Kawasaki Y, Watanabe H, Takahashi R (2015). Induction of potent antitumor immunity by sustained release of cationic antigen from a DNA-based hydrogel with adjuvant activity. Adv. Funct. Mater..

[136] Kim J, Francis DM, Sestito LF, Archer PA, Manspeaker MP (2022). Thermosensitive hydrogel releasing nitric oxide donor and anti-ctla-4 micelles for anti-tumor immunotherapy. Nat. Commun..

[137] Duong HTT, Thambi T, Yin Y, Kim SH, Nguyen TL (2020). Degradation-regulated architecture of injectable smart hydrogels enhances humoral immune response and potentiates antitumor activity in human lung carcinoma. Biomaterials.

[138] Jia YP, Shi K, Yang F, Liao JF, Han RX (2020). Multifunctional nanoparticle loaded injectable thermoresponsive hydrogel as nir controlled release platform for local photothermal immunotherapy to prevent breast cancer postoperative recurrence and metastases. Adv. Funct. Mater..

[139] Sinha A, Choi Y, Nguyen MH, Nguyen TL, Choi SW (2019). A 3d macroporous alginate graphene scaffold with an extremely slow release of a loaded cargo for in situ long-term activation of dendritic cells. Adv. Healthcare Mater..

[140] Yin Y, Li X, Ma H, Zhang J, Yu D (2021). In situ transforming rna nanovaccines from polyethylenimine functionalized graphene oxide hydrogel for durable cancer immunotherapy. Nano Lett..

[141] Thi TTH, Suys EJA, Lee JS, Nguyen DH, Park KD (2021). Lipid-based nanoparticles in the clinic and clinical trials: from cancer nanomedicine to covid-19 vaccines. Vaccines.

[142] Khadke S, Roces CB, Cameron A, Devitt A, Perrie Y (2019). Formulation and manufacturing of lymphatic targeting liposomes using microfluidics. J. Contr. Release.

[143] Oussoren C, Velinova M, Scherphof G, van der Want JJ, van Rooijen N (1998). Lymphatic uptake and biodistribution of liposomes after subcutaneous injection: Iv. Fate of liposomes in regional lymph nodes. Biochim. et Biophys. Acta (BBA) Biomembr..

[144] Chen J, Ye Z, Huang C, Qiu M, Song D (2022). Lipid nanoparticle-mediated lymph node-targeting delivery of mrna cancer vaccine elicits robust cd8(+) t cell response. Proc. Natl. Acad. Sci..

[145] Oussoren C, Zuidema J, Crommelin DJA, Storm G (1997). Lymphatic uptake and biodistribution of liposomes after subcutaneous injection.: Ii. Influence of liposomal size, lipid composition and lipid dose. Biochim. et Biophys. Acta (BBA) Biomembr..

[146] Luozhong S, Yuan Z, Sarmiento T, Chen Y, Gu W (2022). Phosphatidylserine lipid nanoparticles promote systemic rna delivery to secondary lymphoid organs. Nano Lett..

[147] Li X, Wu Y, Wang S, Liu J, Zhang T (2022). Menthol nanoliposomes enhanced anti-tumor immunotherapy by increasing lymph node homing of dendritic cell vaccines. Clinical Immunol..

[148] Stoffel M, Wolfrum C, Shi S, Jayaprakash KN, Jayaraman M (2007). Mechanisms and optimization of in vivo delivery of lipophilic sirnas. Nat. Biotechnol..

[149] Dixon JB (2010). Lymphatic lipid transport: sewer or subway?. Trends Endocrinol. Metab..

[150] Lim HY, Thiam CH, Yeo KP, Bisoendial R, Hii CS (2013). Lymphatic vessels are essential for the removal of cholesterol from peripheral tissues by sr-bi-mediated transport of hdl. Cell Metab..

[151] Wan D, Que H, Chen L, Lan T, Hong W (2021). Lymph-node-targeted cholesterolized TLR7 agonist liposomes provoke a safe and durable antitumor response. Nano Lett..

[152] Papahadjopoulos D (1988). Liposome formation and properties: an evolutionary profile. Biochem. Soci. Trans..

[153] Düzgüneş N, Nir S (1999). Mechanisms and kinetics of liposome–cell interactions. Adv. Drug Deliv. Rev..

[154] Zhai Y, He X, Li Y, Han R, Ma Y (2021). A splenic-targeted versatile antigen courier: Ipsc wrapped in coalescent erythrocyte-liposome as tumor nanovaccine. Sci. Adv..

[155] Kamaly N, Yameen B, Wu J, Farokhzad OC (2016). Degradable controlled-release polymers and polymeric nanoparticles: mechanisms of controlling drug release. Chem. Rev..

[156] Gothwal A, Khan I, Gupta U (2016). Polymeric micelles: recent advancements in the delivery of anticancer drugs. Pharm. Res..

[157] Nishimoto Y, Nagashima S, Nakajima K, Ohira T, Sato T (2020). Carboxyl-, sulfonyl-, and phosphate-terminal dendrimers as a nanoplatform with lymph node targeting. Int. J. Pharm..

[158] Xu Y, Ma S, Zhao J, Chen H, Si X (2022). Mannan-decorated pathogen-like polymeric nanoparticles as nanovaccine carriers for eliciting superior anticancer immunity. Biomaterials.

[159] Wang L, He Y, He T, Liu G, Lin C (2020). Lymph node-targeted immune-activation mediated by imiquimod-loaded mesoporous polydopamine based-nanocarriers. Biomaterials.

[160] Jiang D, Gao T, Liang S, Mu W, Fu S (2021). Lymph node delivery strategy enables the activation of cytotoxic t lymphocytes and natural killer cells to augment cancer immunotherapy. ACS Appl. Mater. Interfaces.

[161] Karabin NB, Allen S, Kwon HK, Bobbala S, Firlar E (2018). Sustained micellar delivery via inducible transitions in nanostructure morphology. Nat. Commun..

[162] Elsabahy M, Wooley KL (2012). Design of polymeric nanoparticles for biomedical delivery applications. Chem. Soci. Rev..

[163] Liu Z, Zhou C, Qin Y, Wang Z, Wang L (2017). Coordinating antigen cytosolic delivery and danger signaling to program potent cross-priming by micelle-based nanovaccine. Cell Discov..

[164] Wang L, Wang Z, Qin Y, Liang W (2020). Delivered antigen peptides to resident cd8α+ dcs in lymph node by micelle-based vaccine augment antigen-specific cd8+ effector t cell response. Eur. J. Pharm. Biopharm..

[165] Schudel A, Chapman AP, Yau M-K, Higginson CJ, Francis DM (2020). Programmable multistage drug delivery to lymph nodes. Nat. Nanotechnol..

[166] Kim H, Niu L, Larson P, Kucaba TA, Murphy KA (2018). Polymeric nanoparticles encapsulating novel TLR7/8 agonists as immunostimulatory adjuvants for enhanced cancer immunotherapy. Biomaterials.

[167] He B, Hu H-Y, Tan T, Wang H, Sun K-X (2018). Ir-780-loaded polymeric micelles enhance the efficacy of photothermal therapy in treating breast cancer lymphatic metastasis in mice. Acta Pharm. Sin..

[168] Yang X, Yu T, Zeng Y, Lian K, Zhou X (2020). Ph-responsive biomimetic polymeric micelles as lymph node-targeting vaccines for enhanced antitumor immune responses. Biomacromol.

[169] Hess KL, Medintz IL, Jewell CM (2019). Designing inorganic nanomaterials for vaccines and immunotherapies. Nano Today.

[170] Gu L (2018). Tailored silica nanoMater for immunotherapy. ACS Cent. Sci..

[171] Hu H, Yang C, Zhang F, Li M, Tu Z (2021). A versatile and robust platform for the scalable manufacture of biomimetic nanovaccines. Adv. Sci..

[172] Stead SO, McInnes SJP, Kireta S, Rose PD, Jesudason S (2018). Manipulating human dendritic cell phenotype and function with targeted porous silicon nanoparticles. Biomaterials.

[173] Algar WR, Prasuhn DE, Stewart MH, Jennings TL, Blanco-Canosa JB (2011). The controlled display of biomolecules on nanoparticles: a challenge suited to bioorthogonal chemistry. Bioconj. Chem..

[174] Pakhomy SS, Bucharskaya AB, Maslyakova GN, Zlobina OV, Bugaeva IO (2019). The influence of long-term peroral administration of gold nanoparticles with various sizes on the liver, spleen, and lymph nodes of laboratory rats and their progeny. Opt. Spectr..

[175] Liu H, Dong H, Zhou N, Dong S, Chen L (2018). Spio enhance the cross-presentation and migration of dcs and anionic spio influence the nanoadjuvant effects related to interleukin-1β. Nanoscale Res. Lett..

[176] Cha BG, Jeong JH, Kim J (2018). Extra-large pore mesoporous silica nanoparticles enabling co-delivery of high amounts of protein antigen and toll-like receptor 9 agonist for enhanced cancer vaccine efficacy. ACS Cent. Sci..

[177] Wagner J, Gößl D, Ustyanovska N, Xiong M, Hauser D (2021). Mesoporous silica nanoparticles as ph-responsive carrier for the immune-activating drug resiquimod enhance the local immune response in mice. ACS Nano.

[178] Gulla SK, Rao BR, Moku G, Jinka S, Nimmu NV (2019). In vivo targeting of DNA vaccines to dendritic cells using functionalized gold nanoparticles. Biomater. Sci..

[179] Ni K, Luo T, Lan G, Culbert A, Song Y (2020). A nanoscale metal–organic framework to mediate photodynamic therapy and deliver CpG oligodeoxynucleotides to enhance antigen presentation and cancer immunotherapy. Angew. Chem. Int. Ed..

[180] Rashed MH, Bayraktar E, Helal GK, Abd-Ellah MF, Amero P (2017). Exosomes: from garbage bins to promising therapeutic targets. Int. J. Mol. Sci..

[181] Zhang L, Yu D (2019). Exosomes in cancer development, metastasis, and immunity. Biochim. Biophys. Acta.

[182] Hood JL (2017). The association of exosomes with lymph nodes. Semin. Cell Dev. Biol..

[183] Ji P, Yang Z, Li H, Wei M, Yang G (2021). Smart exosomes with lymph node homing and immune-amplifying capacities for enhanced immunotherapy of metastatic breast cancer. Mol. Ther. Nucleic Acids.

[184] Phung CD, Pham TT, Nguyen HT, Nguyen TT, Ou W (2020). Anti-ctla-4 antibody-functionalized dendritic cell-derived exosomes targeting tumor-draining lymph nodes for effective induction of antitumor t-cell responses. Acta Biomater..

[185] Yu G, Jung H, Kang YY, Mok H (2018). Comparative evaluation of cell- and serum-derived exosomes to deliver immune stimulators to lymph nodes. Biomaterials.

[186] Hong L, Xu L, Jin L, Xu K, Tang W (2022). Exosomal circular RNA hsa_circ_0006220, and hsa_circ_0001666 as biomarkers in the diagnosis of pancreatic cancer. J. Clin. Lab. Anal..

[187] Chen W, Li G, Li Z, Zhu J, Wei T (2022). Evaluation of plasma exosomal mirnas as potential diagnostic biomarkers of lymph node metastasis in papillary thyroid carcinoma. Endocrine.

[188] Zhu Z, Chen Z, Wang M, Zhang M, Chen Y (2022). Detection of plasma exosomal mirna-205 as a biomarker for early diagnosis and an adjuvant indicator of ovarian cancer staging. J. Ovarian Res..

[189] Srinivasan S, Vannberg FO, Dixon JB (2016). Lymphatic transport of exosomes as a rapid route of information dissemination to the lymph node. Sci. Rep..

[190] Sun B, Zhou Y, Fang Y, Li Z, Gu X (2019). Colorectal cancer exosomes induce lymphatic network remodeling in lymph nodes. Int. J. Cancer.

[191] Broggi MAS, Maillat L, Clement CC, Bordry N, Corthésy P (2019). Tumor-associated factors are enriched in lymphatic exudate compared to plasma in metastatic melanoma patients. J. Exp. Med..

[192] Hoshino A, Costa-Silva B, Shen T-L, Rodrigues G, Hashimoto A (2015). Tumour exosome integrins determine organotropic metastasis. Nature.

[193] Leary N, Walser S, He Y, Cousin N, Pereira P (2022). Melanoma-derived extracellular vesicles mediate lymphatic remodelling and impair tumour immunity in draining lymph nodes. J. Extracell. Vesicles.

[194] Ma G, Wu C (2017). Microneedle, bio-microneedle and bio-inspired microneedle: a review. J. Contr. Release.

[195] Kim Y-C, Park J-H, Prausnitz MR (2012). Microneedles for drug and vaccine delivery. Adv. Drug Deliv. Rev..

[196] Quinn HL, Kearney M-C, Courtenay AJ, McCrudden MTC, Donnelly RF (2014). The role of microneedles for drug and vaccine delivery. Expert Opinion Drug Deliv..

[197] Wu X, Li Y, Chen X, Zhou Z, Pang J (2019). A surface charge dependent enhanced th1 antigen-specific immune response in lymph nodes by transfersome-based nanovaccine-loaded dissolving microneedle-assisted transdermal immunization. J. Mater. Chem. B.

[198] Kim NW, Kim S-Y, Lee JE, Yin Y, Lee JH (2018). Enhanced cancer vaccination by in situ nanomicelle-generating dissolving microneedles. ACS Nano.

[199] Kwon S, Velasquez FC, Rasmussen JC, Greives MR, Turner KD (2019). Nanotopography-based lymphatic delivery for improved anti-tumor responses to checkpoint blockade immunotherapy. Theranostics.

[200] Menon I, Bagwe P, Gomes KB, Bajaj L, Gala R (2021). Microneedles: a new generation vaccine delivery system. Micromachines.

[201] Niu L, Chu LY, Burton SA, Hansen KJ, Panyam J (2019). Intradermal delivery of vaccine nanoparticles using hollow microneedle array generates enhanced and balanced immune response. J. Contr. Release.

[202] Li Z, He Y, Deng L, Zhang Z-R, Lin Y (2020). A fast-dissolving microneedle array loaded with chitosan nanoparticles to evoke systemic immune responses in mice. J. Mater. Chem. B.

[203] Chen G, Chen Z, Wen D, Wang Z, Li H (2020). Transdermal cold atmospheric plasma-mediated immune checkpoint blockade therapy. Proc. Natl. Acad. Sci..

[204] Zeng Q, Gammon JM, Tostanoski LH, Chiu Y-C, Jewell CM (2017). In vivo expansion of melanoma-specific t cells using microneedle arrays coated with immune-polyelectrolyte multilayers. ACS Biomater. Sci. Eng..

[205] Duong HTT, Yin Y, Thambi T, Nguyen TL, Giang Phan VH (2018). Smart vaccine delivery based on microneedle arrays decorated with ultra-ph-responsive copolymers for cancer immunotherapy. Biomaterials.

[206] Jung J, Lim SY, Kim D, Lyu S, Whang O (2022). Microneedle-directed drug delivery to tumor-draining lymph node for synergistic combination chemoimmunotherapy for metastatic cancer. Adv. Ther..

[207] Yang P, Lu C, Qin W, Chen M, Quan G (2020). Construction of a core-shell microneedle system to achieve targeted co-delivery of checkpoint inhibitors for melanoma immunotherapy. Acta Biomater..

[208] Kim H, Seong K-Y, Lee JH, Park W, Yang SY (2019). Biodegradable microneedle patch delivering antigenic peptide–hyaluronate conjugate for cancer immunotherapy. ACS Biomater. Sci. Eng..

[209] Duong HTT, Yin Y, Thambi T, Kim BS, Jeong JH (2020). Highly potent intradermal vaccination by an array of dissolving microneedle polypeptide cocktails for cancer immunotherapy. J. Mater. Chem. B.

[210] He Y, Hong C, Fletcher SJ, Berger AG, Sun X (2022). Peptide-based cancer vaccine delivery via the stingδtm-cgamp complex. Adv. Healthcare Mater..

[211] Mohsen MO, Augusto G, Bachmann MF (2020). The 3ds in virus-like particle based-vaccines: design, delivery and dynamics. Immunol. Rev..

[212] Mohsen MO, Speiser DE, Knuth A, Bachmann MF (2020). Virus-like particles for vaccination against cancer. WIREs Nanomed. Nanobiotechnol..

[213] Cubas R, Zhang S, Kwon S, Sevick-Muraca EM, Li M (2009). Virus-like particle (vlp) lymphatic trafficking and immune response generation after immunization by different routes. J. Immunother..

[214] Ma L, Dichwalkar T, Chang JYH, Cossette B, Garafola D (2019). Enhanced car–t cell activity against solid tumors by vaccine boosting through the chimeric receptor. Science.

[215] Wang P, Zhao P, Dong S, Xu T, He X (2018). An albumin-binding polypeptide both targets cytotoxic t lymphocyte vaccines to lymph nodes and boosts vaccine presentation by dendritic cells. Theranostics.

[216] Liang X, Niu Z, Galli V, Howe N, Zhao Y (2022). Extracellular vesicles engineered to bind albumin demonstrate extended circulation time and lymph node accumulation in mouse models. J. Extracell. Vesicles.

